# Anion Binding by Fluorescent Ureido-Hexahomotrioxacalix[3]arene Receptors: An NMR, Absorption and Emission Spectroscopic Study

**DOI:** 10.3390/molecules27103247

**Published:** 2022-05-19

**Authors:** Alexandre S. Miranda, Paula M. Marcos, José R. Ascenso, Mário N. Berberan-Santos, Filipe Menezes

**Affiliations:** 1 Centro de Química Estrutural, Institute of Molecular Sciences, Faculdade de Ciências, Universidade de Lisboa, Edifício C8, 1749-016 Lisboa, Portugal; miranda.m.alexandre@gmail.com; 2 IBB-Institute for Bioengineering and Biosciences, Instituto Superior Técnico, Universidade de Lisboa, 1049-001 Lisboa, Portugal; 3 Faculdade de Farmácia, Universidade de Lisboa, Av. Prof. Gama Pinto, 1649-003 Lisboa, Portugal; 4 Centro de Química Estrutural, Institute of Molecular Sciences, Instituto Superior Técnico, Complexo I, Av. Rovisco Pais, 1049-001 Lisboa, Portugal; jose.ascenso@ist.utl.pt; 5 Institute of Structural Biology, Helmholtz Zentrum Muenchen, Ingolstaedter Landstr. 1, 85764 Neuherberg, Germany; filipemmenezes@gmail.com

**Keywords:** hexahomotrioxacalix[3]arenes, fluorescent anion receptors, NMR studies, UV–Vis absorption studies, fluorescence studies, theoretical calculations

## Abstract

Fluorescent receptors (**4a**–**4c**) based on (thio)ureido-functionalized hexahomotrioxacalix[3]arenes were synthesised and obtained in the partial cone conformation in solution. Naphthyl or pyrenyl fluorogenic units were introduced at the lower rim of the calixarene skeleton via a butyl spacer. The binding of biologically and environmentally relevant anions was studied with NMR, UV–vis absorption, and fluorescence titrations. Fluorescence of the pyrenyl receptor **4c** displays both monomer and excimer fluorescence. The thermodynamics of complexation was determined in acetonitrile and was entropy-driven. Computational studies were also performed to bring further insight into the binding process. The data showed that association constants increase with the anion basicity, and AcO^−^, BzO^−^ and F^−^ were the best bound anions for all receptors. Pyrenylurea **4c** is a slightly better receptor than naphthylurea **4a**, and both are more efficient than naphthyl thiourea **4b**. In addition, ureas **4a** and **4c** were also tested as ditopic receptors in the recognition of alkylammonium salts.

## 1. Introduction

Fluorescence spectroscopy, due to its high sensitivity and simplicity, is an attractive technique used for the quantitative determination of ions. Lately, a wide range of fluorescence sensors based on calixarenes have been used in various applications, namely in the detection of biologically and environmentally relevant cations and anions [1,2,3]. Owing to their structural features, these macrocycle compounds have been widely investigated as neutral molecule and ion receptors [4,5]. They possess a well-defined hydrophobic cavity available in different sizes and conformations, and an almost unlimited number of derivatives can be obtained by functionalisation of their upper and lower rims. Different fluorophores like naphthalene, anthracene and pyrene are among the most incorporated in the calixarene framework, leading to fluorescent probes for the recognition of different types of analytes. Examples of fluorescent calix[4]arene [6,7,8,9,10,11,12,13], calix[5]arene [14] and calix[6]arene [15,16] receptors have been reported in the literature.

Anions [17,18,19] such as fluoride, chloride, and iodide play important roles in many medical systems, and the carboxylate group is present in several biological molecules. Concerning the environment, the treatment of nitrates, sulphates, phosphates, and perchlorates, among other anions, is also a demanding field. Synthetic anion receptors, namely calixarenes bearing (thio)urea moieties, can form strong and directional hydrogen bonds between their NH groups and the anions. Some of these calixarenes also behave as ditopic receptors [20], simultaneously binding both ions of a given ion pair.

As part of our continuous interest on the host–guest properties of homooxacalixarenes (calixarene analogues in which the CH_2_ bridges are partly or completely replaced by CH_2_OCH_2_ groups) [21,22], we have reported the anion and ion pair recognition of several (thio)ureido-dihomooxacalix[4]arenes [23,24,25], including fluorescent receptors [26,27]. The ion affinity of hexahomotrioxacalix[3]arenes, a very interesting macrocycle formed by an 18-membered ring and having only two basic conformations, has also been investigated [28,29,30,31,32,33]. Recently, we have extended our research into the study of fluorescent hexahomotrioxacalix[3]arenes. Thus, this paper describes the synthesis of three new fluorescent derivatives bearing naphthylurea (**4a**), naphthylthiourea (**4b**), and pyrenylurea (**4c**) residues at the lower rim via a butyl spacer. These compounds were obtained in a partial cone conformation in solution. Their affinity towards relevant anions and alkylammonium salts was assessed by proton NMR, UV–Vis absorption and fluorescence spectroscopy. The thermodynamics of the anion complexation was determined in acetonitrile by absorption and emission, and computational studies were also performed to bring further insight about the binding process.

## 2. Results and Discussion

### 2.1. Synthesis and Conformational Analysis

**Scheme 1:**
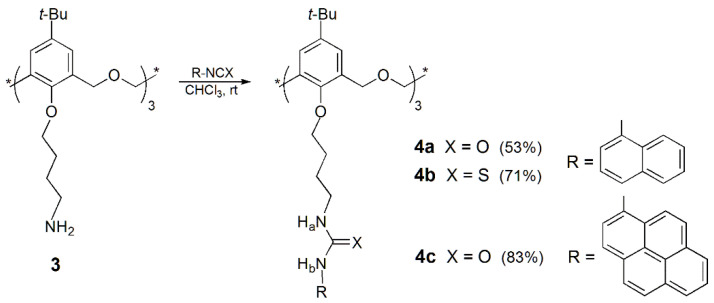
Synthesis of (thio)urea receptors **4a**–**4c**.

Recently, we have reported the synthesis of three hexahomotrioxacalix[3]arene- based receptors bearing (thio)urea groups linked to the macrocycle lower rim by a butyl spacer [32]. Following this line of research, we prepared three new fluorescent sensors containing (thio)urea residues and naphthalene or pyrene moieties. Thus, a three-step procedure (already described) [32] was undertaken from parent compound *p-tert*-butylhexahomotrioxacalix[3]arene **1**. The alkylation reaction of **1** with *N*-(4-bromobutyl)phthalimide and K_2_CO_3_ afforded triphthalimide compound **2**. The phthalimido groups were further removed with hydrazine, yielding triamine **3**, which reacted with naphthyliso(thio)cyanate or 1-pyrenylisocyanate to give the corresponding tri(thio)urea receptors (naphthyl urea **4a**, naphthyl thiourea **4b** and pyrenyl urea **4c**) in the partial cone conformation (Scheme 1).

These derivatives exhibit symmetric proton and carbon-13 NMR spectra compatible with a *C*_s_ symmetry. ^1^H NMR spectra in chloroform at room temperature show two singlets (in a 1:2 ratio) for *tert*-butyl groups, three AB quartets for the CH_2_ bridge protons (CH_2_OCH_2_), one pair of doublets and one singlet for the aromatic protons of the calixarene skeleton, two triplets and two singlets (in a 1:2 ratio) for the NH_a_ and NH_b_ protons, respectively, besides several multiplets for the –OCH_2_CH_2_CH_2_CH_2_N– methylene protons and for the aromatic protons of the naphthyl/pyrenyl groups. The proton assignments were confirmed by COSY spectra. ^13^C NMR spectra show 9 of the expected 10 upfield resonances arising from the *tert*-butyl groups and the methylene carbon atoms of the –OCH_2_***C***H_2_***C***H_2_***C***H_2_N– group, 5 midfield resonances arising from the CH_2_OCH_2_ and the –O***C***H_2_CH_2_CH_2_CH_2_N– groups, and 31 (or 41 in the case of **4c**) downfield resonances arising from the aromatic carbon atoms and the (thio)carbonyl groups. All resonances were assigned by DEPT experiments.

### 2.2. Anion and Ion-Pair Recognition

#### 2.2.1. Proton NMR Studies

The binding properties of (thio)ureas **4a**–**4c** toward several relevant anions of spherical (F^−^, Cl^−^, Br^−^, I^−^), trigonal planar (NO_3_^−^, AcO^−^, BzO^−^) and tetrahedral (HSO_4_^−^, ClO_4_^−^) geometries were investigated using tetrabutylammonium (TBA) salts in CDCl_3_ by proton NMR titrations. The association constants reported as log *K*_ass_ in Table 1 were determined following the NH chemical shifts of the (thio)ureido groups through the WinEQNMR2 programme [34].

**Figure 1:**
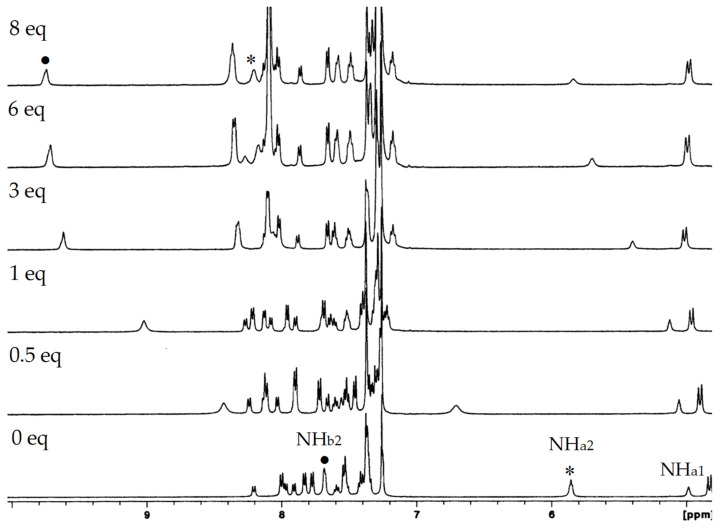
^1^H NMR partial spectra (500 MHz, CDCl_3_, 25 °C) of Naphturea **4a** with several equiv of TBA BzO.

The addition of TBA salts to the receptors resulted in downfield shifts of their NH protons, clearly indicating hydrogen bonding interactions between the (thio)urea groups and the anions (see, for example, Figure 1 for **4a** + BzO^−^). A fast exchange rate between free and complexed receptors was observed on the NMR time scale at room temperature. The titration curves obtained (Figure S1) indicate the formation of 1:1 complexes, this stoichiometry being also confirmed by Job plots (Figure S2).

Data presented in Table 1 show that AcO^−^, BzO^−^ and F^−^ are the best bound anions, and, in general, the association constants increase with increasing of anion basicity for all the receptors. A closer analysis of the results indicates that pyrenyl urea **4c** is a more efficient receptor than naphthyl urea **4a**, presenting association constants that are in average 0.35 log units higher than those obtained for **4a**. This fact may be due to the presence of the bulkier pyrenyl residue that makes receptor **4c** less flexible and consequently more preorganised compared to naphthyl urea **4a**. Concerning spherical halides, ureas **4a** and **4c** both form strong complexes with F^−^ (log *K*_ass_ = 2.91 and 3.31, respectively), and the association constants decrease with decreasing of anion basicity. Complexes with trigonal planar oxoanions AcO^−^ and BzO^−^ display the highest log *K*_ass_ values for **4a** (3.20 and 3.09, respectively) and for **4c** (3.42 and 3.45, respectively), and in the case of the tetrahedral inorganic oxoanion HSO_4_^−^, reasonably good association constants were also obtained.

Naphthyl thiourea **4b** was also studied, and the data reported in Table 1 show that **4b** is a weaker receptor than corresponding urea **4a** despite the increased acidity of its NH groups. Thiourea **4b** displays the same trend as urea **4a**, the anions being bound according to their basicity for all the geometry groups. The association constants were 0.85 log units lower on average than those obtained for **4a**, except for the more basic and best bound anions F^−^, AcO^−^ and BzO^−^ (Δ log *K*_ass_ = 0.18, 0.41 and 0.48, respectively). Identical situations were reported for several homooxacalixarene thiourea receptors [23,27,32]. The larger size of the sulphur atom that distorts the *cis–cis* geometry required for anion binding may be the cause of this effect. As a result, less preorganised and energetically less favourable thiourea groups are obtained compared to the urea ones [35].

The calixarene skeletons of these partial cone ureas seem to undergo only slight conformational changes upon complexation, as observed before for other hexahomotrioxa derivative analogues [32]. Very small chemical shift variations were observed for the *tert*-butyl protons upon the addition of 8 equiv of the salts for both ureas (Δ*δ* ≤ 0.03 ppm). One of the three aromatic protons also showed small downfield variations (Δ*δ* ≤ 0.07 and 0.09 ppm for **4a** and **4c**, respectively), while the others are difficult to follow due to overlapping by the naphthyl/pyrenyl peaks.

Naphthyl and pyrenyl ureas **4a** and **4c** were also tested in the recognition of *n*-propyl and *n*-butylammonium chlorides in a preliminary study to evaluate their ability as ditopic receptors.

Proton NMR titrations were carried out by adding increasing amounts (up to two equiv) of the salts to CDCl_3_ solutions of receptors **4a** and **4c**. In the case of **4a**, the addition of one equiv of both guests at room temperature caused broadening of the peaks; while for **4c** this broadening was also followed by the appearance of resonances at high field. This indicates host–guest interaction with both receptors, and the inclusion of alkyl groups inside the aromatic cavity of host **4c** at room temperature. To better analyse the spectra, the temperature was lowered to 233 K. For both ureas, peaks have appeared in the negative region of the spectra (Figure S3), but they remain broad, suggesting that the complexation/decomplexation process is still fast on the NMR time scale. Given these results, no other ion pairs were tested.

#### 2.2.2. UV–Vis Absorption and Fluorescence Studies

The proton NMR studies of the interactions between (thio)ureas **4a**–**4c** and the previous anions were complemented by UV–Vis absorption and fluorescence titrations in dichloromethane, and also in acetonitrile in the case of urea **4a**.

**Figure 2:**
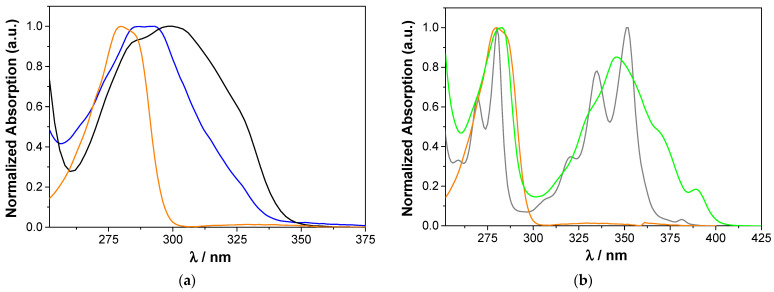
Normalised absorption spectra of (**a**) **1** (orange), **A** (blue) and **4a** (black) and (**b**) **1** (orange), **B** (grey) and **4c** (green) in CH_2_Cl_2_ at 25 °C. [1] = [**A**] = $5\times 10^{-5}\;$ M, [**4a**] = $2\times 10^{-5}\;$ M; [**B**] = $4\times 10^{-5}\;$ M, [**4c**] = $1\times 10^{-5}$ M.

**Figure 3:**
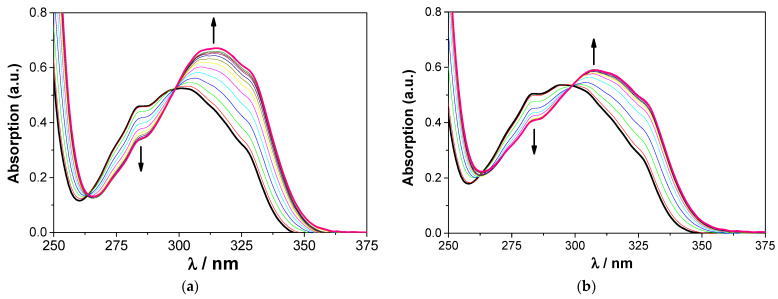
Changes in the UV spectrum of urea **4a** (2.0 × 10^−5^ M) upon addition of TBA F (up to 10 equiv.) in (**a**) CH_2_Cl_2_ and (**b**) MeCN. The arrows indicate the effect of increasing amounts of salt.

Naphthylurea **4a** behaved similarly in both solvents, displaying an absorption band centred at approximately 301 or 295 nm in CH_2_Cl_2_ or MeCN, respectively, but with a marked shoulder at 330 nm and an onset at about 360 nm. The low-energy part of the absorption (wavelengths larger than about 300 nm) mainly arises from the naphthalene moieties, as shown by a comparison with both the spectrum of parent calixarene **1** whose lowest energy absorption is benzenoid, peaking at 282 nm and with an onset at about 300 nm, and with the absorption spectrum of naphthyl precursor **A**, whose absorption peaks at 300 nm, displays a shoulder at 330 nm, and had the onset at about 360 nm (Figure 2a). Upon the addition of increasing amounts of F^−^, Cl^−^, AcO^−^ and BzO^−^, this band decreases in intensity while a new one progressively appears at longer wavelengths. In the case of F^−^, the new maximum is reached at approximately 315 or 308 nm, with red shifts of 14 or 13 nm, respectively in CH_2_Cl_2_ or MeCN. Isosbestic points at 263 and 299 nm are observed in both solvents (Figure 3). For the other anions, similar absorption spectral changes were observed (Figure S4), showing also isosbestic points and red shifts, albeit smaller (from 9 to 6 nm). Concerning Br^−^, NO_3_^−^, HSO_4_^−^ and ClO_4_^−^ anions, their complexation by receptor **4a** induced successive increases of the absorption (except for Br^−^ in MeCN that decreases as the anion concentration increase), but with no wavelength shift of the absorption maximum (Figure S5). Naphthyl thiourea **4b** showed a different behaviour for all the anions. In the case of F^−^, two isosbestic points at approximately 277 and 309 nm can be seen, but no significant shift of the absorption maximum is observed (Figure S6a). For the other anions, the absorption band decreases as the anion concentration increases, presenting an isosbestic point around 325 nm (Figure S6b).

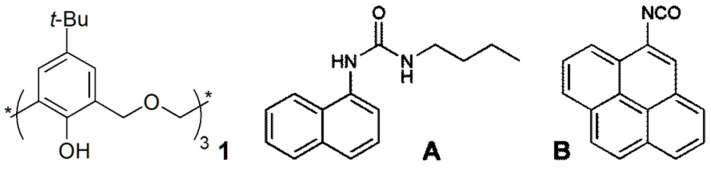



**Figure 4:**
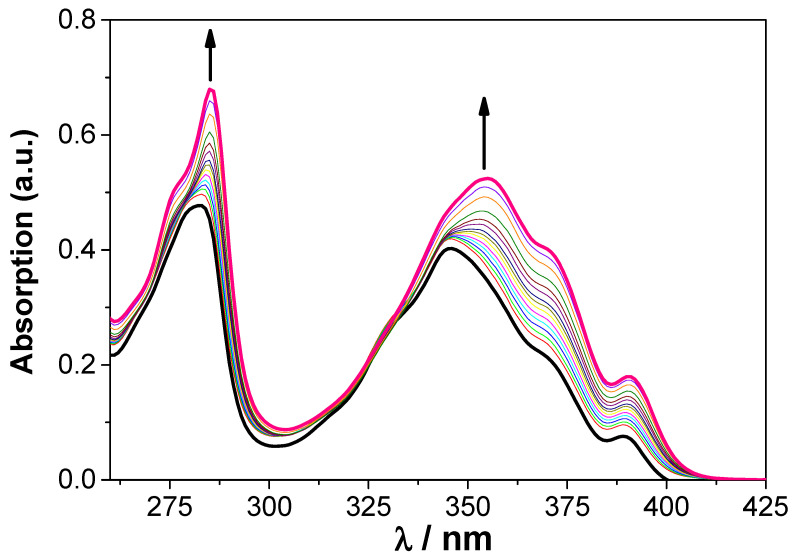
Changes in UV spectrum of urea **4c** (1.0 × 10^−5^ M) upon the addition of TBA AcO (up to 10 equiv.) in CH_2_Cl_2_. Arrows indicate increasing amounts of salt.

Free pyrenyl urea **4c** displays two absorption bands of similar intensity peaking at approximately 283 and 346 nm, both arising from the pyrenyl groups, as shown by comparison with the absorption of the isolated moiety precursor **B** (Figure 2b) and again considering the benzenoid absorption characteristics of the calixarene backbone. The titration of **4c** with F^−^, Cl^−^, AcO^−^ and BzO^−^ anions resulted in an increase of the intensity of both bands with slight bathochromic shifts of their absorption maxima (Figure 4). These shifts were higher for the long wavelength pyrenyl band (Δλ = 12 nm for F^−^ and 4 nm for Cl^−^) compared to the macrocycle one (Δλ = 3 nm for F^−^ and 1 nm for Cl^−^). Additions of Br^−^, NO_3_^−^, HSO_4_^−^ and ClO_4_^−^ anions to **4c** also induced progressive increases in the absorption bands, but no shifts in their maxima were observed (Figure S7).

**Figure 5:**
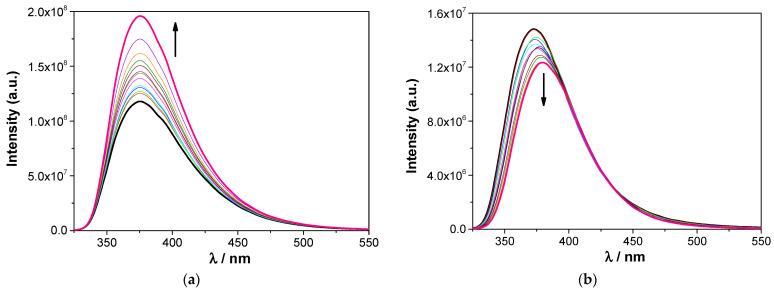
Changes in the emission spectrum of urea **4a** (2.0 × 10^−5^ M) upon addition of TBA F (up to 10 equiv) in (**a**) CH_2_Cl_2_ and (**b**) MeCN. The arrows indicate the effect of increasing amounts of salt.

Naphthylurea **4a** presents emission bands centred at approximately 375 nm in both solvents, as well as nanosecond fluorescence lifetimes and significant quantum yields (Table 2 and Figure S8). No intramolecular excimer is observed in the fluorescence spectra, pointing to the impossibility of naphthyl group pairs to attain near-sandwich configurations. An increase of the emission intensity was obtained upon the addition of all anions to **4a** in CH_2_Cl_2_ (Figure 5a), which was less pronounced for NO_3_^−^, HSO_4_^−^ and ClO_4_^−^ (Figure S9a). In MeCN, an increase of the emission intensity was also observed in the case of Cl^−^, Br^−^, AcO^−^, BzO^−^ and ClO_4_^−^ anions, whereas a quenching of the fluorescence intensity was observed for F^−^ (Figure 5b), NO_3_^−^ and HSO_4_^−^ (Figure S9b). The lifetimes of **4a** either do not change or change moderately upon complexation (Table S1). At 10 equivalents, AcO^−^, BzO^−^ and F^−^have the strongest effect on the lifetimes of **4a**, in agreement with the expected associated fractions (see Figure S10). The observed changes both in lifetimes and in intensities (hence fluorescence quantum yields) probably resulted from conformational modifications mainly affecting the radiative constants.

The fluorescence of thiourea **4b** could not be studied, as this derivative is unstable upon irradiation.

**Figure 6:**
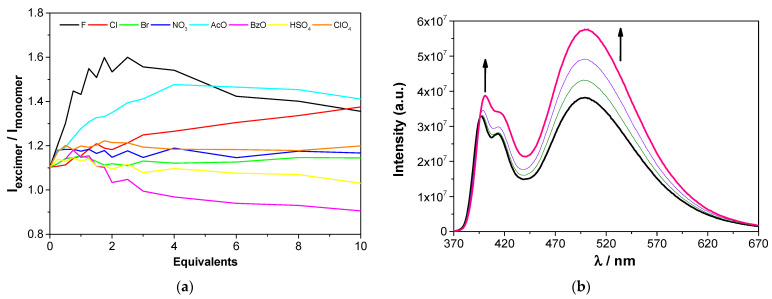
(**a**) Ratio of excimer and monomer peak intensities of urea **4c** upon addition of TBA salts, (**b**) Changes in the emission spectrum of urea **4c** upon addition of 0 (black), 0.75 (olive), 2 (violet) and 10 equiv. (pink) of TBA AcO. The arrows indicate the increasing amounts of salt. [**4c**] = 1.0 × 10^−5^ M in CH_2_Cl_2_.

The fluorescence of **4c** exhibits both monomer (ca. 400 nm) and excimer (ca. 500 nm) bands arising from the pyrene moieties (Table 2 and Figure S8). The overall fluorescence quantum yield is significant, and monomer and excimer have very different lifetimes, the average monomer lifetime being of the order of 2 ns, significantly shorter than that of reference compound **B** (Table 2), whereas the excimer lifetime is one order of magnitude higher. Owing to the close proximity of pyrene pairs, the excimer displays a very short risetime (Table S2), indicating that its formation is quite fast. This component is also found in the monomer decay (Table S2) along with a component close to that of reference compound **B**. This probably results from the fact that two pyrenyl units engage in excimer formation, whereas the third, on account of the partial cone conformation, will exhibit isolated monomer behavior. The monomer–excimer double emission, with its associated excited-state kinetics and photophysics, precludes a clear-cut evaluation of association constants from fluorescence. The average monomer lifetimes of **4c** do not change significantly upon complexation (Table S2). It is nevertheless observed that complexation has a varying effect on excimer photophysics depending on the anion (Figure 6a), showing that different conformations of the pyrenylurea arms can be adopted upon complexation. It is observed that in some cases the excimer/monomer intensity ratio is essentially unaffected by anion binding (Figure S11), whereas in other cases there is a strong effect (Figure 6b).

In all cases, significant spectral variations were observed for the three receptors, allowing the determination of the corresponding binding constants by absorption in dichloromethane (Table 3). For naphthyl urea **4a**, the constants were also determined in dichloromethane and acetonitrile, and using both absorption and fluorescence. The association constants obtained for all receptors are on average one log unit higher than those obtained by NMR, but follow approximately the same trend. The more dilute solutions used in the UV–Vis/fluorescence titrations favour the dissociation of the salts, providing a higher concentration of the anions available for complexation and resulting in higher association constants [14]. The data reported in Table 3 show a stronger binding in dichloromethane than that in acetonitrile for urea **4a**, both by absorption and emission, in agreement with the relative competitive character of the two solvents. As observed before (NMR studies), pyrenyl urea **4c** is a slightly better receptor than naphthyl urea **4a** for all the anions, while naphthyl thiourea **4b** is the weakest receptor.

#### 2.2.3. Temperature Dependence of the Association Constants—Thermodynamic Analysis and Simulation

**Figure 7:**
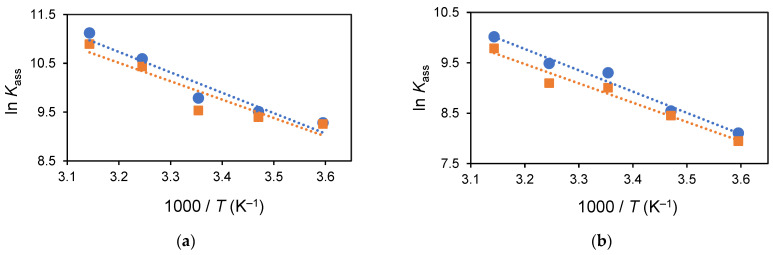
Linear regression of ln *K*_ass_ vs. 1000/*T* using UV–Vis absorption (circles) and fluorescence (squares) for urea **4a** in MeCN (2.0 × 10^−5^ M) upon addition up to 10 equiv of (**a**) TBA F and (**b**) TBA AcO.

The thermodynamic parameters of the complexation of naphthylurea **4a** with F^−^ and AcO^−^ in acetonitrile were determined from the dependence of the association constant with temperature using the van’t Hoff equation (Figure 7 and Table 4). According to Table 4, similar results were obtained from both experimental techniques. A general conclusion drawn from the data is that the complexation is entropy-driven in the two cases studied, which is surprising and was only observed once in similar systems [36].

**Figure 8:**
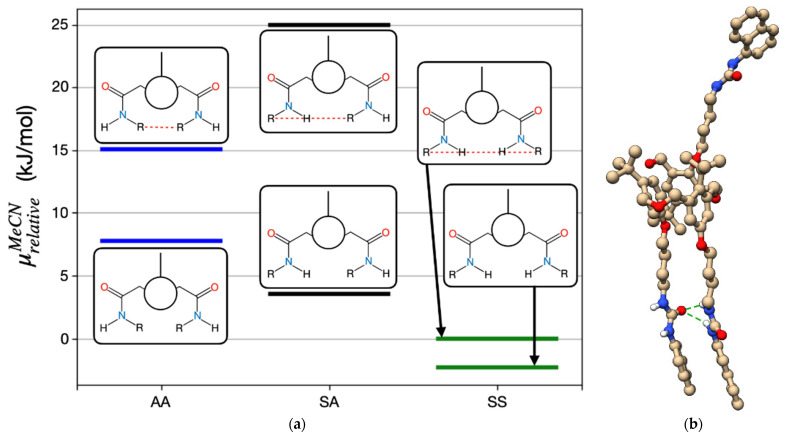
(**a**) Relative chemical potentials of **4a** arm main conformers in MeCN (as a continuum dielectric) at 300 K. Dashed lines denote π-stacking interactions of the naphthyl groups; (**b**) optimised structure for the SS’ conformer. Intramolecular hydrogen bonds are represented by green dashed lines.

To better understand the entropy effect, extensive simulations were carried out. For this purpose, several main conformations of naphthylurea **4a** arms (oriented on the same side of the macrocyclic ring) were identified, according to the orientation of each urea NH and naphthyl moieties. Each urea group may exist in a *sin* (S, both protons in the same direction) or in an *anti* (A) orientation, and the naphthyl groups may be π -stacked (structures marked with a prime or identified by dashed lines, as shown in Figure 8a) or free (unprimed structures). Figure 8a shows the relative chemical potentials of these species in acetonitrile solutions, and in Figure 8b is represented the optimised structure for the SS’ conformer. Gas phase thermodynamic data is provided in the Supporting Information.

As expected, π -stacked structures are not as stable in solution as the respective open forms, mainly due to the reduced solvent accessible surface areas. The exception is conformer SS’, which benefits from intramolecular hydrogen bonding as well (see Figure 8b). The open form of conformer SA is also reasonably close in chemical potential to conformers SS and SS’. SA’ on the other hand, is quite unstable in solution. Unless there are kinetic factors hindering the interconversion between different species, equilibrium solutions of the calixarene are expected to be dominated by SS and SS’, with a combined mole fraction of 95%. The two SA conformers have a weight of 4%, whereas the AA corresponds to only 1%. The combined estimation of weights is based on the conformational search performed with the program CREST, which showed that primed and unprimed species belong to the same ensemble.

**Figure 9:**
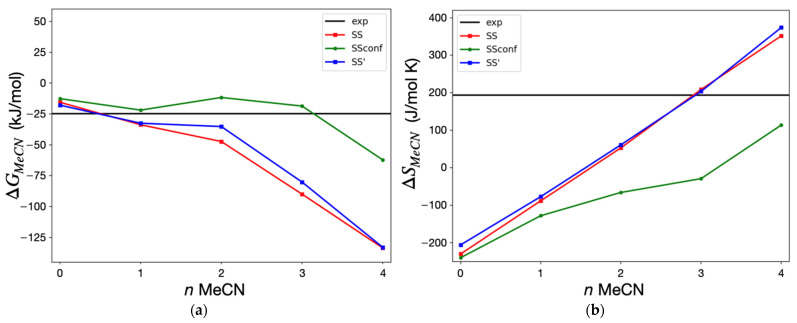
Variation in (**a**) Gibbs energy and (**b**) entropy for the binding F^−^ to **4a** with *n* molecules (*n* = 0–4) of MeCN explicitly considered. The reactions considered to generate the plot are summarised by, e.g., $SS:nMeCN+F^{-}⇌\;nMeCN+SS:F^{-}$ .

Calculated Gibbs energy variations upon binding of F^−^ anion in acetonitrile for conformers SS are displayed in Figure 9a. This figure shows results where the solvent participation is implicit (*n* = 0), as well as hybrid models, where 1 to 4 molecules of acetonitrile are explicitly included in the system (besides the implicit effects from the generalised Born model). These molecules are initially attached to the urea moieties by hydrogen bonding. Calculated gas-phase enthalpies (Figure S12a) and entropies (Figure 9b) significantly differed from experimental data, stressing the role of the solvent. The gas-phase calculations predict enthalpies from −123 up to −83 kJ mol^−1^ and entropy variations also negative from −206 to −230 J mol^−1^ K^−1^ for conformers SS, as one could expect for a binding process. The experimental average values are positive and of the order of 33 kJ mol^−1^ and 195 J mol^−1^ K^−1^, respectively (Table 4). The results for the entropy variations considering an estimation of the conformational effects for conformer SS are also shown in Figure 9b (denoted as SSconf). These results evidence the relevance of considering the loss of conformational degrees of freedom when the calixarene binds the anion.

The designed hybrid models help to clarify the role of acetonitrile in the binding process. Instead of binding, the formation of adducts between fluoride and **4a** is transformed into exchange reactions. Acetonitrile may bind the calixarene by hydrogen bonding or dispersion. Though the enthalpy gain for the latter type of interaction is weaker, entropic penalties for both binding modes are similar. Irrespective of the conformer considered in the binding process, enthalpies and entropies progressively increase with the number of added acetonitrile molecules. On the other hand, Gibbs energies progressively decrease when acetonitrile molecules are explicitly included.

The obtained results clearly show that the explicit inclusion of acetonitrile solvent (as a few discrete molecules) brings the computed enthalpy and entropy closer to the measured values. Furthermore, inclusion of conformational effects is essential for the correct prediction of the entropy changes, keeping this state function from reaching unrealistically large values. The positive enthalpies and entropies observed experimentally result therefore from the direct involvement of the solvent in the binding process.

## 3. Experiments

### 3.1. General Information

All chemicals were reagent-grade and used without further purification. Chromatographic separations were performed on Merck silica gel 60 (particle size 40–63 μm, 230–400 mesh). Melting points were measured (not corrected) on a Stuart Scientific apparatus, and FTIR spectra were recorded on a Shimadzu Model IRaffinity-1 spectrophotometer. ^1^H and ^13^C NMR spectra were recorded on a Bruker Avance III 500 MHz spectrometer with TMS as internal reference. Conventional COSY 45 experiments were conducted as 256 × 2 K complex points. Elemental analysis was determined on a Fisons EA 1108 microanalyser.

### 3.2. Procedure for the Synthesis of Ureas ***4a*** and ***4c***, and Thiourea ***4b***

To a solution of amine **3** [32] (0.53 g, 0.67 mmol) in 30 mL of CHCl_3_ was added 2.01 mmol of the appropriate iso(thio)cyanate. In the case of 1-pyrenylisocyanate, it was synthesised from 1-pyrene amine in the presence of triethylamine and triphosgene, according to reference [37]. The mixture was stirred at room temperature under N_2_ for 4 h. Evaporation of the solvent yielded the crude products that were purified as described below.

*7,15,23-Tri-tert-butyl-25,26,27-tri[[(**N′**-1-naphthylureido)butyl]oxy]-2,3,10,11,18,19-hexahomo-3,11,19-trioxacalix[3]arene* (**4a**): Flash chromatography (SiO_2_, eluent CH_2_Cl_2_/MeOH, from 99.5:0.5 to 99:1); it was obtained in 53% yield (0.46 g); m.p. 157–159 °C; IR (KBr) 3343 cm^−1^ (NH), 1647 cm^−1^ (CO); ^1^H NMR (CDCl_3_, 500 MHz) *δ* 0.68 (m, 4H, OCH_2_C***H***_2_C***H***_2_CH_2_NH_a_ inverted), 1.25 [s, 18H, C(CH_3_)_3_], 1.30 [s, 9H, C(CH_3_)_3_ inverted], 1.39 (m, 4H, OCH_2_CH_2_C***H***_2_CH_2_NH_a_), 1.53, 1.64 (2m, 4H, OCH_2_C***H***_2_CH_2_CH_2_NH_a_), 2.83 (m, 4H, OC***H***_2_CH_2_CH_2_C***H***_2_NH_a_ inverted), 3.23 (m, 4H, OCH_2_CH_2_CH_2_C***H***_2_NH_a_), 3.40, 3.56 (2m, 4H, OC***H***_2_CH_2_CH_2_CH_2_NH_a_), 4.14, 4.69 (ABq, 4H, *J* = 12.1 Hz, CH_2_OCH_2_), 4.20, 4.28 (Abq, 4H, *J* = 11.0 Hz, CH_2_OCH_2_), 4.44, 4.83 (Abq, 4H, *J* = 11.4 Hz, CH_2_OCH_2_), 4.99 (t, 1H, NH_a_ inverted), 5.89 (t, 2H, NH_a_), 7.25, 7.36 (2d, 4H, ArH), 7.32–7.36 (m, 4H, NH_b_-inverted and Napht), 7.37 (s, 2H, ArH inverted), 7.40, 7.59 (2t, 4H, Napht), 7.50–7.54 (d + t, 4H, Napht), 7.68, 7.76, 7.82, 7.90, 7.96, 7.99, 8.20 (7d, 10H, Napht), 7.71 (s, 2H, NH_b_); NMR ^13^C (CDCl_3_, 125.8 MHz) *δ* 25.0, 26.3 (OCH_2_CH_2_***C***H_2_CH_2_NH_a_), 27.77, 27.80 (OCH_2_***C***H_2_CH_2_CH_2_NH_a_), 31.4, 31.5 [C(***C***H_3_)_3_], 34.31, 34.33 [***C***(CH_3_)_3_], 40.0 (2C) (OCH_2_CH_2_CH_2_***C***H_2_NH_a_), 64.0, 66.3, 68.4 (CH_2_OCH_2_), 74.8, 75.3 (O***C***H_2_CH_2_CH_2_CH_2_NH_a_), 119.8, 120.9, 121.3, 124.40, 124.43, 125.8, 125.9 (3C), 126.0, 126.1, 126.2, 127.4, 127.6, 128.5, 128.7, 128.8 (ArH), 127.5, 127.6, 129.4, 130.1, 130.3, 133.9, 134.2 (2C), 134.4, 146.3, 146.6, 155.3 (Ar), 156.1, 157.2 (CO). Anal. Calcd for C_81_H_96_N_6_O_9_: C 74.97; H 7.46; N 6.48. Found: C 75.04; H 7.82; N 6.24.

*7,15,23-Tri-tert-butyl-25,26,27-tri[[(**N′**-1-naphthylthioureido)butyl]oxy]-2,3,10,11,18,19-hexahomo-3,11,19-trioxacalix[3]arene* (**4b**): Flash chromatography (SiO_2_, eluent CH_2_Cl_2_/MeOH, from 99.9:0.1 to 99.3:0.7); it was obtained in 71% yield (0.64 g); m.p. 132–134 °C; ^1^H NMR (CDCl_3_, 500 MHz) δ 0.56 (m, 2H, OCH_2_C**H**_2_CH_2_CH_2_NH_a_ inverted), 0.74 (m, 2H, OCH_2_CH_2_CH_2_CH_2_NH_a_ inverted), 1.23 [s, 9H, C(CH_3_)_3_ inverted], 1.24 [s, 18H, C(CH_3_)_3_], 1.37 (m, 8H, OCH_2_CH_2_C**H**_2_CH_2_NH_a_), 2.38 (m, 2H, OC**H**_2_CH_2_CH_2_CH_2_NH_a_ inverted), 2.91 (m, 2H, OCH_2_CH_2_CH_2_CH_2_NH_a_ inverted), 3.36 (m, 4H, OC**H**_2_CH_2_CH_2_CH_2_NH_a_), 3.42 (m, 4H, OCH_2_CH_2_CH_2_CH_2_NH_a_), 3.72, 4.36 (Abq, 4H, J = 10.9 Hz, CH_2_OCH_2_), 3.73, 3.99 (Abq, 4H, J = 11.0 Hz, CH_2_OCH_2_), 3.99, 4.44 (Abq, 4H, J = 10.1 Hz, CH_2_OCH_2_), 5.97 (broad t, 1H, NH_a_ inverted), 6.41 (broad t, 2H, NH_a_), 7.07 (broad s, 4H, ArH), 7.12 (s, 2H, ArH inverted), 7.43 (d, 2H, Napht), 7.47–7.52 (m, 8H, Napht), 7.57 (m, 2H, Napht), 7.73 (s, 2H, NH_b_), 7.74 (s, 1H, NH_b_ inverted), 7.83–7.88 (m, 5H, Napht), 7.90–7.94 (m, 3H, Napht), 8.05 (d, 1H, Napht); ^13^C NMR (CDCl_3_, 125.8 MHz) δ 24.2, 25.2 (OCH_2_CH_2_**C**H_2_CH_2_NH_a_), 27.4, (OCH_2_**C**H_2_CH_2_CH_2_NH_a_), 31.5, 31.6 [C(**C**H_3_)_3_], 34.2 [**C**(CH_3_)_3_], 44.7, 45.3 (OCH_2_CH_2_CH_2_**C**H_2_NH_a_), 64.2, 66.3, 68.5 (CH_2_OCH_2_), 73.4, 74.7 (O**C**H_2_CH_2_CH_2_CH_2_NH_a_), 122.4, 122.6, 125.2, 125.3, 125.6, 125.7, 126.9, 127.0, 127.3, 127.38, 127.44, 127.8, 128.3, 128.56, 128.59, 128.62 (ArH), 129.5, 130.0, 130.2, 130.9, 132.3, 132.5, 134.6, 145.8, 145.9, 154.4, 155.2 (Ar), 181.6, 181.8 (CS). Anal. Calcd for C_81_H_96_N_6_O_6_S_3_: C 72.29; H 7.19; N 6.24; S 7.15. Found: C 71.87; H 7.16; N 6.21; S 6.86.

*7,15,23-Tri-tert-butyl-25,26,27-tri[[(**N′**-1-pyrenylureido)butyl]oxy]-2,3,10,11,18,19-hexahomo-3,11,19-trioxacalix[3]arene* (**4c**)*:* Recrystallization from CH_2_Cl_2_/*n*-hexane; it was obtained in 83% yield (0.85 g); m.p. 191–193 °C; IR (KBr) 3318 cm^−1^ (NH), 1638 cm^−1^ (CO); ^1^H NMR (CDCl_3_, 500 MHz) *δ* 0.74 (m, 4H, OCH_2_C***H***_2_C***H***_2_CH_2_NH_a_ inverted), 1.26 [s, 18H, C(CH_3_)_3_], 1.36 [s, 9H, C(CH_3_)_3_ inverted], 1.48 (m, 4H, OCH_2_CH_2_C***H***_2_CH_2_NH_a_), 1.57, 1.78 (2m, 4H, OCH_2_C***H***_2_CH_2_CH_2_NH_a_), 2.84 (t, 2H, OC***H***_2_CH_2_CH_2_CH_2_NH_a_ inverted), 2.92 (m, 2H, OCH_2_CH_2_CH_2_C***H***_2_NH_a_ inverted), 3.19, 3.42 (2m, 4H, OCH_2_CH_2_CH_2_C***H***_2_NH_a_), 3.46, 3.62 (2m, 4H, OC***H***_2_CH_2_CH_2_CH_2_NH_a_), 4.15, 4.31 (Abq, 4H, *J* = 11.1 Hz, CH_2_OCH_2_), 4.19, 4.84 (Abq, 4H, *J* = 11.5 Hz, CH_2_OCH_2_), 4.49, 4.86 (Abq, 4H, *J* = 11.5 Hz, CH_2_OCH_2_), 5.03 (t, 1H, NH_a_ inverted), 5.94 (t, 2H, NH_a_), 7.26, 7.43 (2d, 4H, ArH), 7.42 (s, 2H, ArH inverted), 7.48, 7.60 (2d, 4H, Pyr), 7.67 (m, 3H Pyr), 7.73–7.80 (m, 4H, Pyr), 7.79 (s, 1H, NH_b_ inverted), 7.85–7.92 (m, 4H, Pyr), 7.89 (s, 2H, NH_b_), 7.98–8.06, 8.10–8.18 (m, 9H, Pyr), 8.22, 8.42, 8.55 (3d, 3H, Pyr); ^13^C NMR (CDCl_3_, 125.8 MHz) *δ* 25.0, 26.4 (OCH_2_CH_2_***C***H_2_CH_2_NH_a_), 27.7, 27.8 (OCH_2_***C***H_2_CH_2_CH_2_NH_a_), 31.4, 31.6 [C(***C***H_3_)_3_], 34.3, 34.4 [***C***(CH_3_)_3_], 40.1 (2C) (OCH_2_CH_2_CH_2_***C***H_2_NH_a_), 64.0, 66.4, 68.5 (CH_2_OCH_2_), 74.7, 75.2 (O***C***H_2_CH_2_CH_2_CH_2_NH_a_), 120.3, 120.5, 121.5, 121.6, 124.1, 124.6, 124.7, 124.8, 125.3, 125.56, 125.63, 125.7, 126.2, 126.4, 126.7, 126.9, 127.4, 127.5, 127.7, 127.8, 128.8 (ArH), 123.4, 124.2, 124.7, 125.0, 125.5, 127.9, 128.2, 129.4, 130.1, 130.3, 130.4, 131.0, 131.4, 131.5, 146.4, 146.6, 155.2 (Ar), 156.3, 157.5 (CO). Anal. Calcd for C_99_H_102_N_6_O_9_: C 78.23; H 6.76; N 5.53. Found: C 78.17; H 6.40; N 5.75.

### 3.3. ^1^H NMR Titrations

The anion association constants (as log *K*_ass_) were determined in CDCl_3_ by ^1^H NMR titration experiments. Several aliquots (up to 10 equiv.) of the anion solutions (as TBA salts) were added to 0.5 mL solution of the receptors (2.5 × 10^−3^ M) directly in the NMR tube. The spectra were recorded after each addition of the salts, and the temperature of the NMR probe was kept constant at 25 °C. The association constants were evaluated using the WinEQNMR2 program [34] and following the urea NH chemical shifts. The Job methods were performed keeping the total concentration in 2.5 × 10^−3^ M, the same concentration used in the ion-pair recognition studies.

### 3.4. UV–Vis Absorption and Fluorescence Studies

Absorption and fluorescence studies were conducted using a Shimadzu UV-3101PC UV-Vis–NIR spectrophotometer and a Fluorolog F112A fluorimeter in right-angle configuration, respectively. Association constants were determined in CH_2_Cl_2_ and MeCN by UV–Vis absorption spectrophotometry and by steady-state fluorescence at 25 °C. Absorption spectra were recorded between 250 and 430 nm, and the emission ones between 320 and 670 nm, and using quartz cells with an optical path length of 1 cm. Several aliquots (up to 10 equiv.) of the anion solutions (as TBA salts) were added to a 2 mL solution of the receptors (1.0 × 10^−5^ − 2.0 × 10^−5^ M) directly in the cell. Emission spectra were corrected for the spectral response of the optics and the photomultiplier. Fluorescence quantum yields were measured using quinine sulphate as the reference (*Φ*_F_ = 0.546 in H_2_SO_4_ 0.5 M). Time-resolved fluorescence intensity decays were obtained using the single-photon timing method with laser excitation and microchannel plate detection, with the already described setup [38]. The excitation wavelengths used were at the maximum absorption of the calixarenes and the emission wavelengths at maximum emission, using a front-face geometry. The timescale varied from 12.2 to 24.4 ps per channel for derivative **4a,** and 12.2 to 136.7 ps per channel for derivative **4c**. The spectral changes were interpreted using the HypSpec 2014 program [39]. The thermodynamic parameters were obtained under the same conditions in a temperature range from 5 to 45 °C. Details concerning the photophysical properties determination were already provided [25].

### 3.5. Computational Details

Geometry optimisations and thermodynamics were calculated using the newly developed C++ library, ULYSSES [40]. The method of choice was GFN2-xTB [41] using ALPB for solvation effects [42]. The initial structures of the calixarene were generated with Avogadro [43,44]. Geometries were minimised using the BFGS algorithm along with the dogleg trust region with convergence criteria of $5\times 10^{-8}\;E_{h}$ for energies and $2.5\times 10^{-5}\;E_{h}/a_{0}$ for gradients. The method of Lindh et al. was used to approximate the Hessian matrix in the step calculation [45]. As the intramolecular π -stacking interactions of naphthyl groups are extremely favorable in the gas phase, the resulting geometries were not faithful representations of the system in solution. Therefore, the structures were always optimised in the dielectric of acetonitrile. Further details are given in the Supporting Information.

## 4. Conclusions

The anion binding properties of three fluorescent (thio)ureido-hexahomotrioxacalix[3]arene receptors were investigated by NMR, UV–Vis absorption and fluorescence titrations. These derivatives, bearing naphthyl or pyrenyl moieties at the macrocycle lower rim linked by a butyl spacer, were obtained in the partial cone conformation in solution. Anions of different geometries were bound through hydrogen bonds in a 1:1 stoichiometry. The results showed that for all the receptors the association constants increase with the anion basicity and the carboxylates AcO^−^ and Bzo^−^, and the halide F^−^ were the best bound anions. Pyrenyl urea **4c** is a more efficient receptor than naphthyl urea **4a**, as shown by all the spectroscopic methods used. This may be due to the presence of the bulkier pyrenyl moiety that makes **4c** less flexible and consequently more preorganised. Fluorescence of **4c** displays both monomer and excimer fluorescence. The thermodynamics data obtained in acetonitrile for **4a** with F^−^ and AcO^−^ anions indicated that the binding process is entropy-driven. The solvation of this type of macrocyclic skeleton, with three oxygen bridges (CH_2_OCH_2_), is expected to be stronger than that of a dihomooxacalix[4]arene, for example, and may explain the low enthalpy and the high entropy terms obtained [36]. Computational studies performed showed the critical role of the solvent on the anion-receptor association, approaching the computed enthalpy and entropy to the experimental values. As ditopic receptors, ureas **4a** and **4c** both showed some ion pair recognition, but at low temperature, the complexation/decomplexation process is still fast on the NMR time scale.

## Figures and Tables

**Table 1:** Association constants (log *K*_ass_) ^a^ of hexahomotrioxa (thio)ureas **4a–4c** determined by ^1^H NMR in CDCl_3_ at 25 °C.

	Spherical				Trigonal Planar			Tetrahedral	
	F^−^	Cl^−^	Br^−^	I^−^	NO_3_^−^	AcO^−^	BzO^−^	HSO_4_^−^	ClO_4_^−^
I. Radius/Å ^b^	1.33	1.81	1.96	2.20	1.79	2.32	—	1.90	2.00
Napht urea **4a**	2.91	2.88	2.33	1.83	2.29	3.20	3.09	2.70	1.57
Napht thiourea **4b**	2.73	2.00	1.23	1.11	1.40	2.79	2.61	2.05	1.18
Pyr urea **4c**	3.31	3.19	2.78	2.23	2.67	3.42	3.45	2.97	1.99

^a^ Estimated error ˂ 10%. ^b^ Data quoted in I. Marcus, *Ion Properties*, Marcel Dekker, New York, pp. 50–51, 1997.

**Table 2:** Photophysical properties of model compounds **A** and **B** and ureas **4a** and **4c** at 25 °C.

	Solvent	*λ*_max,abs_ (nm)	*ε* (M^−1^ cm^−1^)	*λ*_max,em_ (nm)	Stokes Shift ^a^ (nm)	*τ*_f_ (ns)	*Φ*_F_^b^	*k*_r_ (ns^−1^)	*k*_nr_ (ns^−1^)
**A**	CH_2_Cl_2_	293	$2.5\times 10^{4}$	372	79	2.61	0.52	0.20	0.18
**B**	CH_2_Cl_2_	352	$4.7\times 10^{4}$	412	60	5.70	0.14	0.025	0.15
**4a**	CH_2_Cl_2_	301	$5.1\times 10^{4}$	375	74	4.84	0.37	0.076	0.13
	MeCN	295	$5.6\times 10^{4}$	374	79	3.67	0.35	0.095	0.18
**4c**	CH_2_Cl_2_	283 ^c^	$5.6\times 10^{4}$	398 ^e^	115	1.98 ^g^	0.19 ^i^	―	―
		340 ^d^		498 ^f^	158	25.9 ^h^		―	―

^a^ Computed as $\lambda_{\max,\mathrm{em}}-\lambda_{\max,\mathrm{abs}}$ ; ^b^ Against quinine sulfate *Φ*_F_ = 0.546 in H_2_SO_4_ 0.5 M; ^c^ Abs_max_ (calixarenic macrocycle + pyrene band); ^d^ Absmax (pyrene band); ^e^ Corresponding to the monomer band; ^f^ corresponding to the excimer band; ^g^ $\lambda_{\mathrm{em}}=$ 398 nm; ^h^ $\lambda_{\mathrm{em}}=$ 498 nm; ^i^ overall (monomer + excimer) emission.

**Table 3:** Association constants (log *K*_ass_)^a^ of (thio)ureas **4a**–**4c** determined by UV–Vis absorption and emission at 25 °C.

			Spherical			Trigonal Planar			Tetrahedral	
		Solvent	F^−^	Cl^−^	Br^−^	NO_3_^−^	AcO^−^	BzO^−^	HSO_4_^−^	ClO_4_^−^
**4a**	Abs	CH_2_Cl_2_	4.34	3.74	3.43	3.51	4.23	4.28	3.65	2.69
		MeCN	4.25	3.51	3.26	3.28	4.04	4.01	3.54	2.54
	Emi	CH_2_Cl_2_	4.20	3.78	3.29	3.44	4.07	4.13	3.45	2.65
		MeCN	4.14	3.46	3.18	3.19	3.91	4.06	3.29	2.48
**4b**	Abs	CH_2_Cl_2_	3.39	3.00	2.78	2.62	3.28	3.19	2.91	2.26
**4c**	Abs	CH_2_Cl_2_	4.46	3.97	3.65	3.60	4.29	4.37	3.80	2.92

^a^ Estimated error ˂ 10%.

**Table 4:** Thermodynamic quantities for the complexation of anions with **4a** in MeCN at 25 °C determined by UV–Vis and fluorescence.

	log *K*_ass_	Δ*G*° (kJ mol^−1^)	Δ*H*° (kJ mol^−1^)	Δ*S*° (J mol^−1^K^−1^)
F^−^	4.25/4.14	−25/−25	35/32	200/190
AcO^−^	4.04/3.91	−23/−22	35/32	190/180

## Data Availability

Not applicable.

## Supplementary Materials

The following are available online: https://www.mdpi.com/1420-3049/27/10/3247/s1. Titration curves with TBA salts in CDCl_3_; Job’s plots; absorption and emission spectra with TBA salts; computational details; ^1^H, ^13^C, DEPT and COSY NMR spectra. References [46,47,48,49,50,51,52,53,54] are cited in the supplementary.

## Sample Availability

Samples of the compounds are not available from the authors.
