# Correction: Miranda et al. Anion Binding by Fluorescent Ureido-Hexahomotrioxacalix[3]arene Receptors: An NMR, Absorption and Emission Spectroscopic Study. *Molecules* 2022, *27*, 3247

**DOI:** 10.3390/molecules29081892

**Published:** 2024-04-22

**Authors:** Alexandre S. Miranda, Paula M. Marcos, José R. Ascenso, Mário N. Berberan-Santos, Filipe Menezes

**Affiliations:** 1 Centro de Química Estrutural, Institute of Molecular Sciences, Faculdade de Ciências, Universidade de Lisboa, Edifício C8, 1749-016 Lisboa, Portugal; miranda.m.alexandre@gmail.com; 2 IBB-Institute for Bioengineering and Biosciences, Instituto Superior Técnico, Universidade de Lisboa, 1049-001 Lisboa, Portugal; 3 Faculdade de Farmácia, Universidade de Lisboa, Av. Prof. Gama Pinto, 1649-003 Lisboa, Portugal; 4 Centro de Química Estrutural, Institute of Molecular Sciences, Instituto Superior Técnico, Complexo I, Av. Rovisco Pais, 1049-001 Lisboa, Portugal; jose.ascenso@ist.utl.pt; 5 Institute of Structural Biology, Helmholtz Zentrum Muenchen, Ingolstaedter Landstr. 1, 85764 Neuherberg, Germany; filipemmenezes@gmail.com

## Error in Figure

In the original publication [1], there was a mistake in Figure 7. Figure 7a,b incorrectly show the same plot (which was placed twice by mistake). The corrected version of Figure 7a,b appears below. The authors apologize for any inconvenience caused and state that the scientific conclusions are unaffected. This correction was approved by the Academic Editor. The original publication has also been updated.

## Figures and Tables

**Figure 7:**
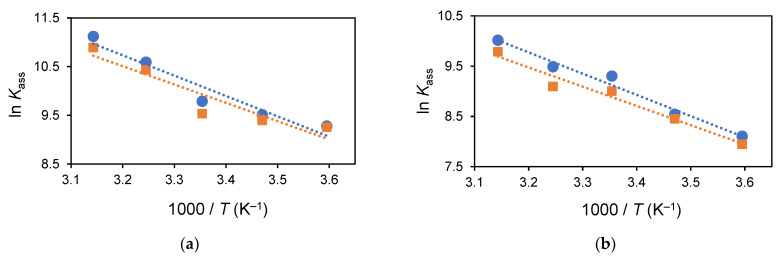
Linear regression of ln *K*_ass_ vs. 1000/*T* using UV–Vis absorption (circles) and fluorescence (squares) for urea **4a** in MeCN (2.0 × 10^−5^ M) upon addition up to 10 equiv of (**a**) TBA F and (**b**) TBA AcO.
