# Supplementary material for: Anion Binding by Fluorescent Ureido-Hexahomotrioxacalix[3]arene Receptors: An NMR, Absorption and Emission Spectroscopic Study
Source: Molecules. 2022 May 19;27(10):3247. doi: 10.3390/molecules27103247 (PMC9142983; doi:10.3390/molecules27103247)
Supplement: Supplementary file 1 [file molecules-27-03247-s001.zip › molecules-1730924-supplementary.pdf]

Supporting Information

Anion Binding by Fluorescent Ureido-Hexahomotrioxacalix[3]arene Receptors: a NMR, Absorption and Emission Spectroscopic Study

Alexandre S. Miranda, Paula M. Marcos,* José R. Ascenso,
Mário N. Berberan-Santos,* Filipe Menezes

List of contents

1. Titration curves of Napht (**4a**) and Pyr (**4c**) ureas with TBA salts in $CDCl_3$ 2
2. Job's plots based on ^{1}H NMR data 3
3. ^{1}H NMR spectra of Napht urea **4a** with *n*-$BuNH_2 \cdot HCl$ in $CDCl_3$ 4
4. Absorption spectra of (thio)ureas with TBA salts in CH_2Cl_2 and MeCN 5
5. Emission spectra of model compounds and ureas with TBA salts in CH_2Cl_2 6
6. Two- and three-exponential analysis of fluorescence decays of **4a** and **4c** with TBA salts 9
7. Computational details 10
8. Electronic supplementary information for computed structures 13
9. ^{1}H NMR spectra of **4a**, **4b** and **4c** 14
10. ^{13}C NMR spectra of **4a**, **4b** and **4c** .17
11. DEPT spectra of **4a**, **4b** and **4c** 20
12. COSY spectra of **4a**, **4b** and **4c** 23

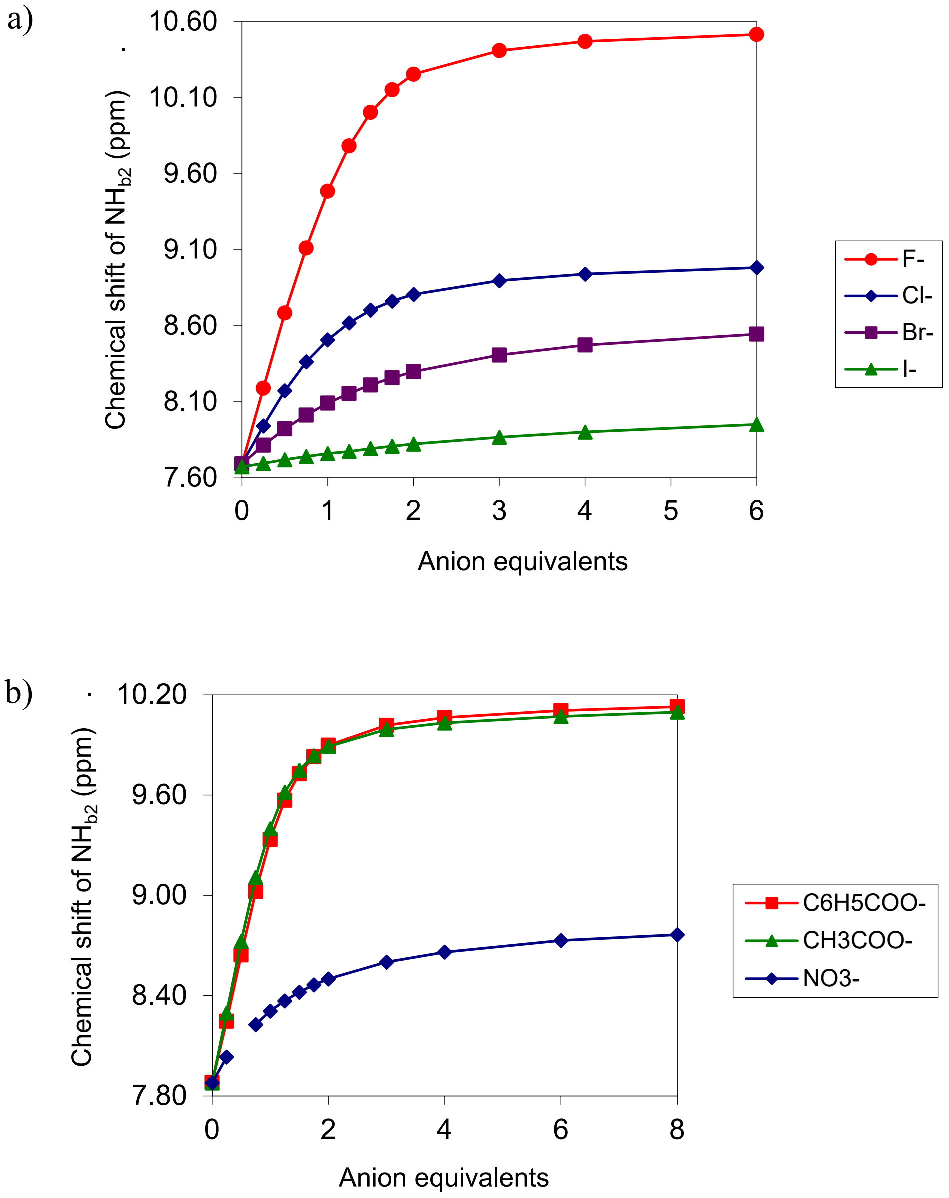

Figure S1. Titration curves of (a) Napht urea **4a** and (b) Pyr urea **4c** with TBA salts in $CDCl_3$.

(a)

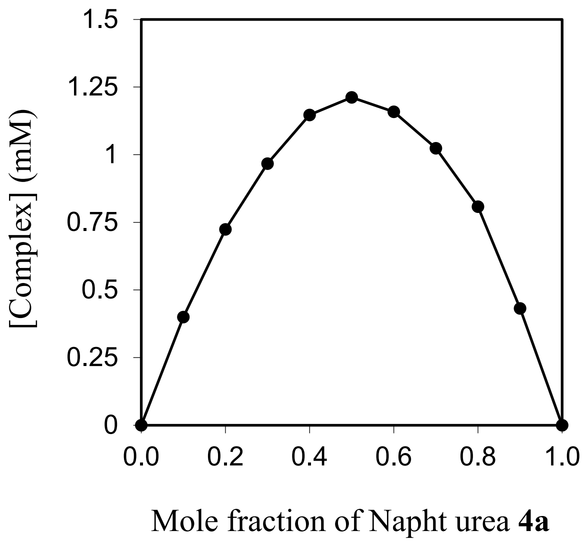

(b)

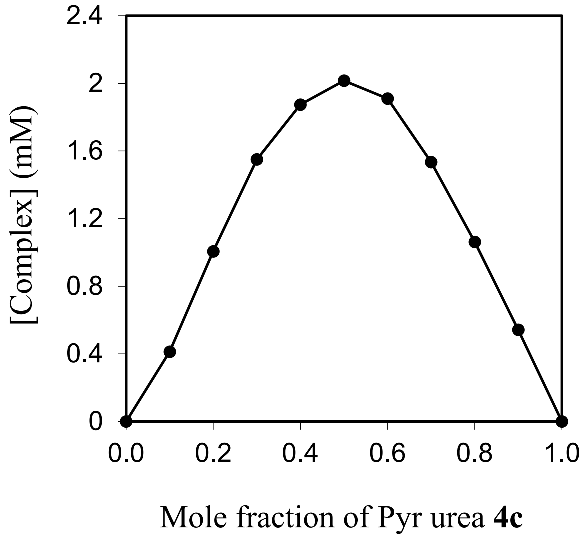

Figure S2. Job plot based on ^{1}H NMR data for (a) Napht urea **4a** + Br^{-}, (b) Pyr urea **4c** + BzO^{-}; total concentration 2.5×10^{-3} M in $CDCl_3$.

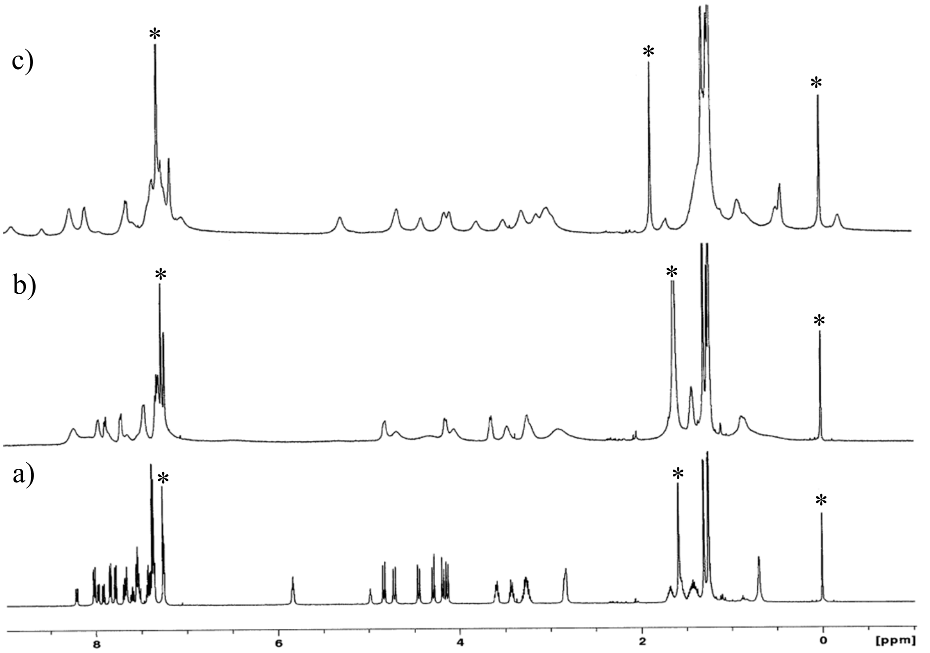

Figure S3. ^{1}H NMR spectra (500 MHz, $CDCl_3$) of: a) Napht urea **4a** at 298 K, b) **4a** + 1 eq of *n*-$BuNH_2{\cdot}HCl$ at 298 K, c) **4a** + 1 eq of *n*-$BuNH_2{\cdot}HCl$ at 233 K. *Denotes residual solvent signals.

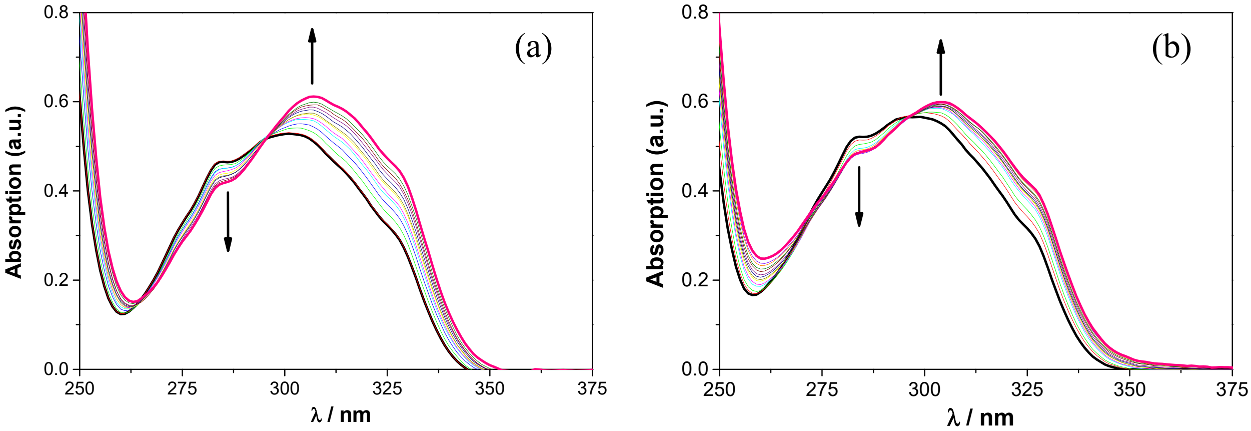

Figure S4. Changes in the UV spectrum of urea **4a** (2.0×10^{-5} M) upon addition of TBA AcO (up to 10 equiv) in (a) CH_2Cl_2 and (b) MeCN. The arrows indicate the effect of increasing amounts of salt.

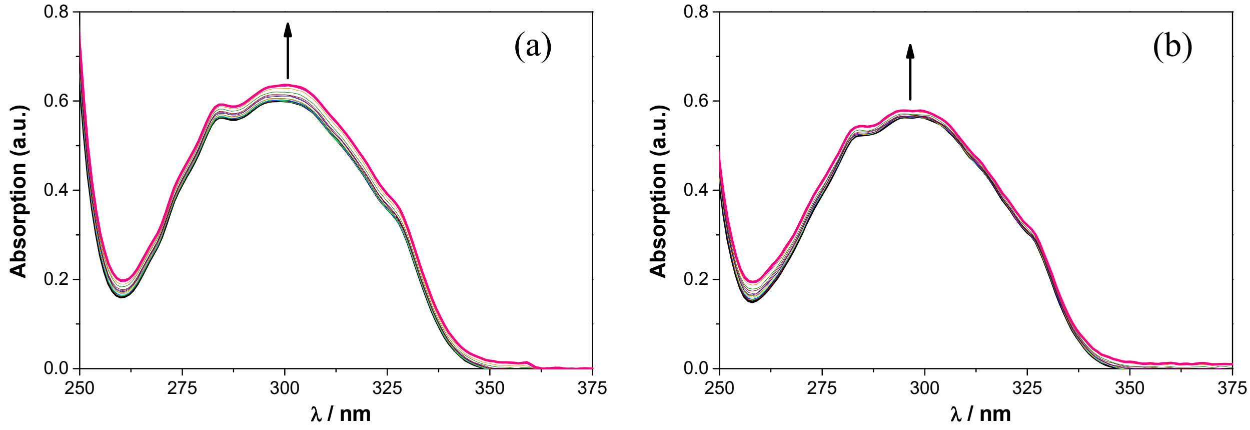

Figure S5. Changes in the UV spectrum of urea **4a** (2.0×10^{-5} M) upon addition of TBA HSO_4 (up to 10 equiv) in (a) CH_2Cl_2 and (b) MeCN. The arrows indicate the increasing amounts of salt.

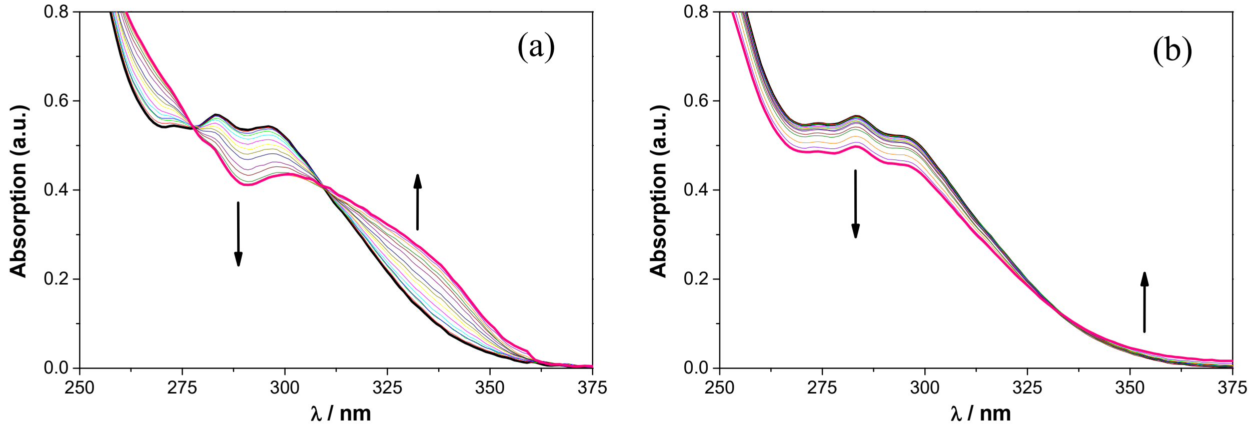

Figure S6. Changes in the UV spectrum of thiourea **4b** (1.0×10^{-5} M) upon addition of (a) TBA F and (b) TBA HSO_4 (up to 10 equiv) in CH_2Cl_2. The arrows indicate the effect of increasing amounts of salt.

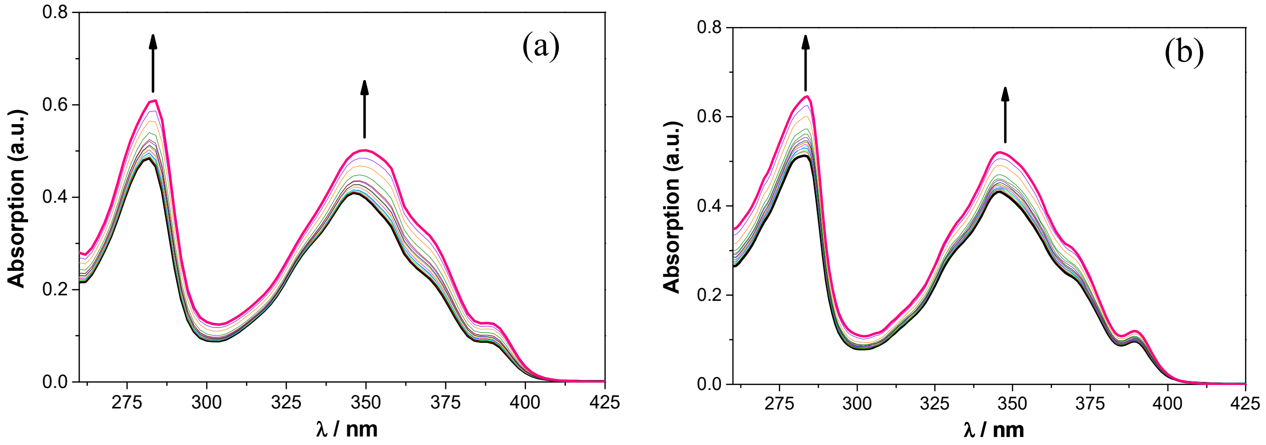

Figure S7. Changes in the UV spectrum of urea **4c** (1.0×10^{-5} M) upon addition of (a) TBA Cl and (b) TBA HSO_4 (up to 10 equiv.) in CH_2Cl_2. The arrows indicate the increasing amounts of salt.

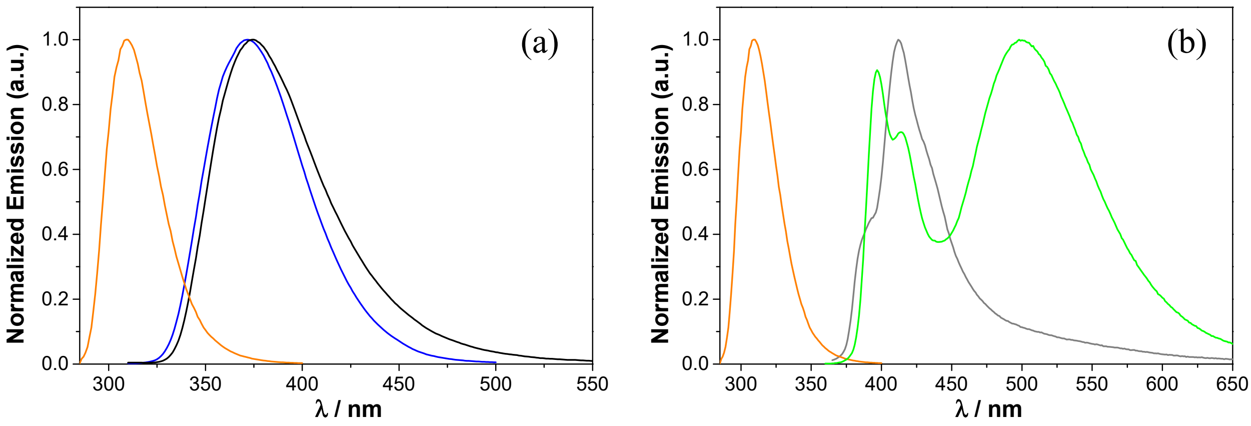

Figure S8: Normalized emission spectra of (a) **1** (orange), **A** (blue) and **4a** (black) and (b) **1** (orange), **B** (grey) and **4c** (green) in CH_2Cl_2 at 25º C. [**1**] = 5×10^{-5}M, $\lambda_{ex} = 280$ nm; [**A**] = 5×10^{-5}M, $\lambda_{ex} = 300$ nm; [**4a**] = 2×10^{-5}M, $\lambda_{ex} = 301$ nm; [**B**] = 4×10^{-5}M, $\lambda_{ex} = 340$ nm; [**4c**] = 1×10^{-5}M, $\lambda_{ex} = 340$ nm.

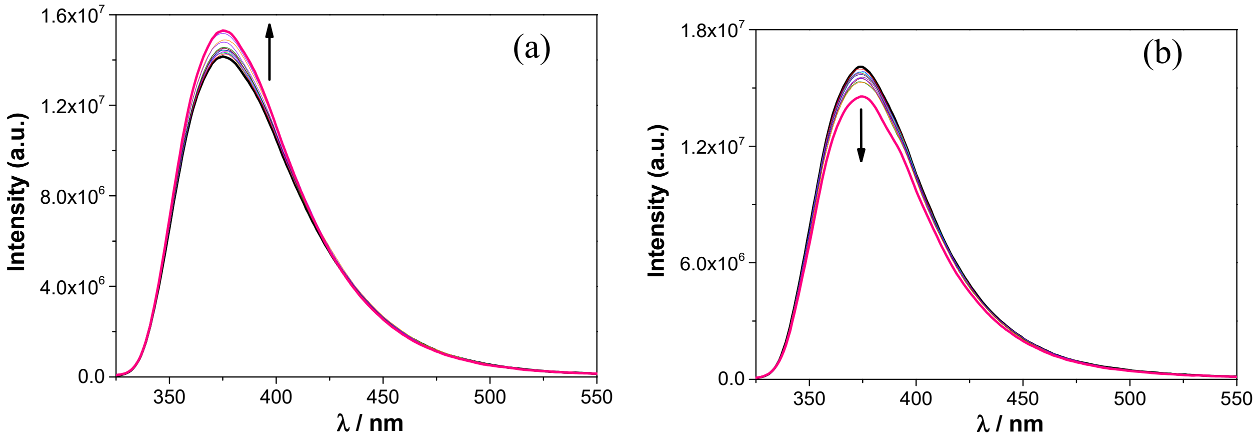

Figure S9. Changes in the emission spectrum of urea **4a** (2.0×10^{-5} M) upon addition of TBA HSO_4 (up to 10 equiv.) in (a) CH_2Cl_2 and (b) MeCN. The arrows indicate the effect of increasing amounts of salt.

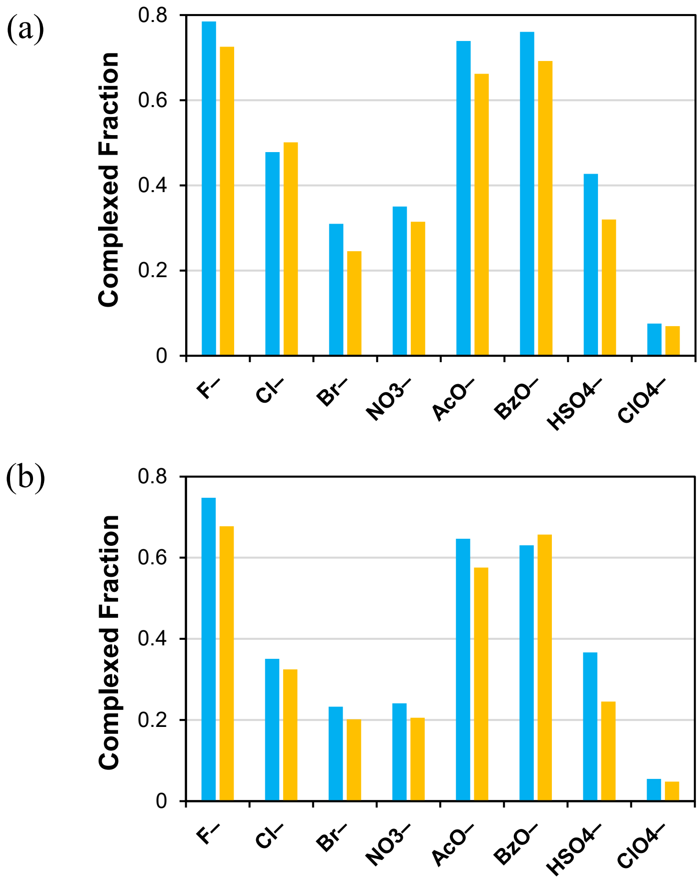

Figure S10. Bound fraction of **4a** (2.0×10^{-5} M) computed using UV-vis absorption (cian) and fluorescence emission (orange) data, in the presence of 10 equiv of TBA salts in (a) CH_2Cl_2 and (b) MeCN.

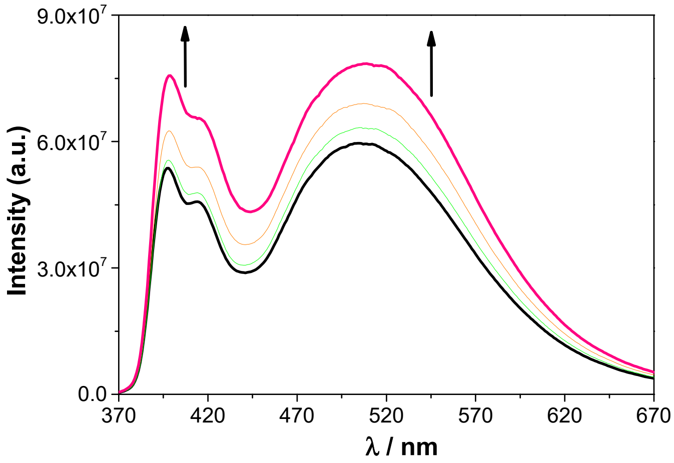

Figure S11. Changes in the emission spectrum of urea **4c** (1.0×10^{-5} M) upon addition of 0 (black), 1 (green), 4 (orange) and 10 equiv. (pink) of TBA HSO_4 in CH_2Cl_2. The arrows indicate the increasing amounts of salt.

Table S1. Two- and three-exponential analysis of fluorescence decays of **4a** with 10 equiv of TBA salts in CH_2Cl_2 and MeCN at 25° C

	CH_2Cl_2				MeCN			
	τ_1/ns (%)	τ_2/ns (%)	τ_3/ns (%)	$\bar{\tau}$/ns	τ_1/ns (%)	τ_2/ns (%)	τ_3/ns (%)	$\bar{\tau}$/ns
**4a**	0.33 (6)	2.6 (59)	9.3 (35)	4.8	3.4 (6)	2.4 (52)	5.6 (42)	3.7
**4a** + F^-	1.5 (12)	3.33 (88)		3.1	1.6 (79)	3.9 (21)		2.1
**4a** + Cl^-	0.62 (4)	2.9 (66)	9.9 (30)	4.9	0.67 (6)	2.7 (60)	7.7 (34)	4.2
**4a** + Br^-	0.35 (5)	2.6 (60)	8.1 (35)	4.4	0.55 (6)	2.5 (54)	5.7 (40)	3.6
**4a** + NO_3^-	0.39 (6)	2.6 (59)	8.8 (35)	4.7	0.55 (6)	2.5 (56)	5.9 (38)	3.7
**4a** + AcO^-	1.6 (64)	3.6 (36)		2.3	1.2 (53)	3.3 (47)		2.2
**4a** + BzO^-	0.54 (25)	1.4 (47)	4.6 (28)	2.1	0.41 (17)	1.3 (46)	3.6 (37)	2.0
**4a** +HSO_4^-	0.40 (6)	2.7 (62)	8.9 (32)	4.5	0.53 (6)	2.5 (55)	5.7 (39)	3.6
**4a** + ClO_4^-	0.46 (6)	2.8 (63)	9.2 (31)	4.6	3.6 (6)	2.5 (56)	5.8 (38)	3.7

Table S2. Two- and three-exponential analysis of fluorescence decays of **4c** with 10 equiv of TBA salts in CH_2Cl_2 at 25° C

	Monomer [a]				Excimer [b]			
	τ_1/ns (%)	τ_2/ns (%)	τ_3/ns (%)	$\bar{\tau}$/ns	τ_1/ns (%)	τ_2/ns (%)	τ_3/ns (%)	$\bar{\tau}$/ns
**4c**	0.32 (18)	0.83 (53)	4.8 (29)	1.9	0.87 (–0.5)	25.9 (100)		25.9
**4c** + F^-	0.16 (26)	0.66 (48)	3.5 (26)	1.3	2.2 (–3)	13.7 (59)	23.8 (44)	18.5
**4c** + Cl^-	0.29 (19)	0.77 (54)	3.9 (27)	1.5	0.89 (–0.1)	23.6 (100)		23.6
**4c** + Br^-	0.28 (15)	0.76 (56)	3.8 (29)	1.6	0.64 (–0.1)	13.5 (11)	26.4 (89)	25.0
**4c** + NO_3^-	0.42 (29)	0.95 (45)	4.7 (26)	1.8	1.2 (–0.2)	25.1 (100)		25.1
**4c** + AcO^-	0.32 (22)	0.81 (52)	4.2 (26)	1.6	0.80 (–0.2)	23.0 (100)		23.0
**4c** + BzO^-	0.25 (17)	0.73 (56)	3.8 (27)	1.5	1.0 (–0.7)	15.6 (18)	24.9 (83)	23.4
**4c** + HSO_4^-	0.46 (31)	1.2 (44)	4.5 (25)	1.8	0.72 (–0.1)	25.4 (100)		25.4
**4c** + ClO_4^-	0.32 (17)	0.80 (55)	4.4 (28)	1.7	1.1 (–0.4)	24.0 (100)		24.0

[a] λ_{ex} = 398 nm; [b] λ_{ex} = 498 nm

Computational Details

Thermodynamic properties were obtained using the free-rotor/harmonic oscillator approximation introduced by Grimme for the calculation of entropies,[1] which was extended for other thermodynamic properties. This is important because the harmonic oscillator approximation is expected to fail for low-frequency vibrational modes, characteristic of non-bonded complexes and flexible molecules. In the ULYSSES program, the interpolation frequency required by this model takes the default value of 75 cm^{-1}. The calculation of conformational entropies was performed with the program CREST,[2] in conjunction with GFN2-xTB and GFN-FF.[3] However, the GFN2-xTB results were used because the ensembles generated from GFN-FF were inadequate and not comparable to the results obtained with GFN2-xTB. To reduce the computational cost, the inverted arm of **4a** was excluded from the calculations. In metadynamics based conformational searches there is often a separation of van der Waals complexes. In order to estimate the conformational entropy of the final product, the number of structures that would allow binding to fluoride were counted from the first 100 conformers and this sub-ensemble was used to estimate the conformational entropy of the whole ensemble collected from CREST (494 conformers + 1143 rotamers). It was assumed that fluoride binding would not affect the relative energies of the conformers in the ensemble. The estimation of the effects of acetonitrile on the conformational ensemble was performed using combinatorics and assuming that binding to acetonitrile would also not affect the relative energies of conformers.

In a few cases it was not possible to obtain adduct geometries without imaginary vibrational frequencies. The workaround was to use the absolute value of the respective imaginary vibrational frequencies in the calculation of thermodynamic properties as suggested by Sure and Grimme.[4] In any case, structures with more than one imaginary frequency larger than 20i cm^{-1} were not accepted. Absolutely no vibrational frequency was disregarded in any part of this work. Plots were generated with python's matplotlib.[5] In order to verify the results obtained with GFN2-xTB, the binding energies were recalculated using PM6-D3H+[6-9] with and without additional corrections from the Axilrod-Teller-Muto three-body dispersion.

(1). Grimme, S. Supramolecular binding thermodynamics by dispersion-corrected density functional theory. *Chem. Eur. J.* **2012**, 18, 9955-9964.

(2). Pracht, P.; Bohle, F.; Grimme, S. Automated exploration of the low-energy chemical space with fast quantum chemical methods. *Phys. Chem. Chem. Phys.* **2020**, 14, 7169-7192.

(3). Spicher, S.; Grimme, S. Robust atomistic modeling of materials, organometallic, and biochemical systems. *Angew. Chem.* **2020**, 59, 15665.
(4). Sure, R.; Grimme, S. Comprehensive benchmark of association (free) energies of realistic host–guest complexes. *J. Chem. Theory Comput.* **2015**, 11, 3785-3801.
(5). Hunter, J. D. Matplotlib: A 2D graphics environment. *Comput. Sci. Eng.* **2007**, *9*, 90-95.
(6). Stewart, J. J. P. Optimization of parameters for semiempirical methods V: Modification of NDDO approximations and application to 70 elements. *J. Mol. Model.* **2007**, *13*, 1173-1213.
(7). Korth, M. Third-generation hydrogen-bonding corrections for semiempirical QM methods and force fields. *J. Chem. Theory Comput.* **2010**, *6*, 3808-3816.
(8). Kromann, J. C.; Christensen, A. S.; Steinmann, C.; Korth, M.; Jensen, J. H. A third-generation dispersion and third-generation hydrogen bonding corrected PM6 method: PM6-D3H+. *Peer J.* **2014**, *2*, 449.
(9). Grimme, S.; Antony, J.; Ehrlich, S.; Krieg, H. A consistent and accurate ab initio parametrization of density functional dispersion correction (DFT-D) for the 94 elements H-Pu. *J. Chem. Phys.* **2010**, *132*, 154104.

(a)

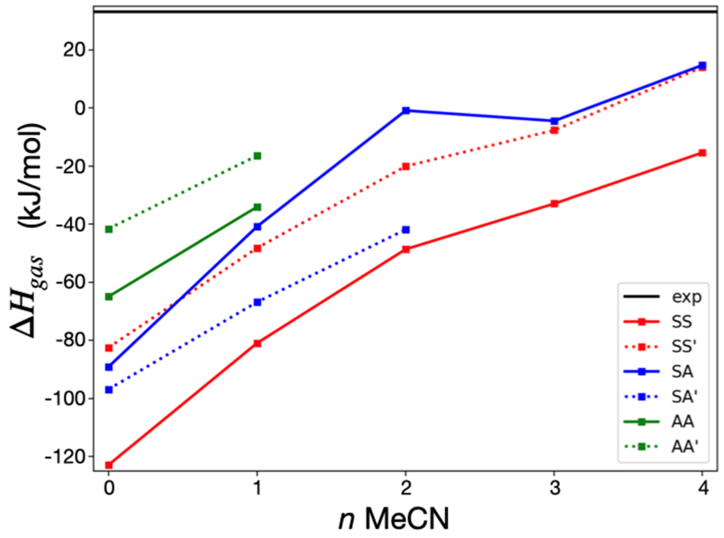

(b)

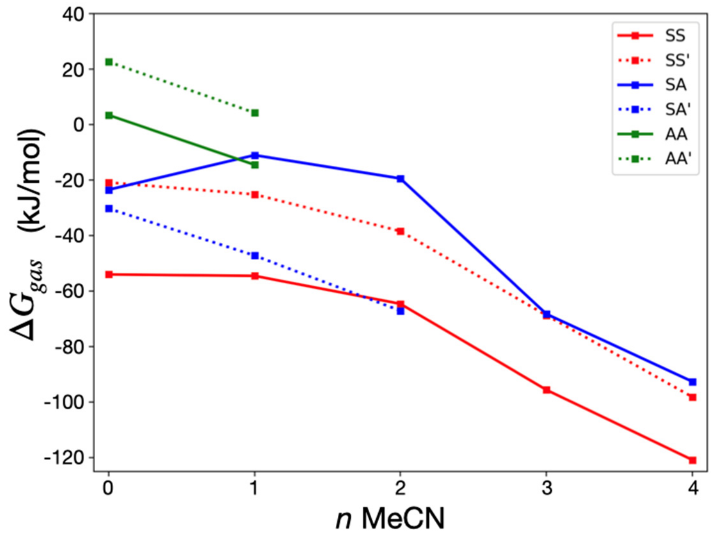

Figure S12. Calculated gas phase enthalpies (a) and Gibbs energies (b) for the exchange reactions defined in the main text (see caption of Figure 9). Note that potential solvation effects are considered only via explicit solvation and no implicit solvation is included.

Electronic supplementary information for computed structures

Cartesian coordinates and energies

See separated file

Object	Integral [rel]	ν(F1) [ppm]
Integral 1	1.0000	8.2130
Integral 2	4.0304	7.9541
Integral 3	6.9558	7.7590
Integral 4	5.1007	7.5489
Integral 5	10.8832	7.3688
Integral 6	1.8103	5.8868
Integral 7	0.9200	5.0013
Integral 8	1.9724	4.8250
Integral 9	1.9269	4.7012
Integral 10	1.9005	4.4460
Integral 11	5.9336	4.2059
Integral 12	1.9594	3.5606
Integral 13	2.0832	3.4067
Integral 14	3.9529	3.2454
Integral 15	3.9012	2.8252
Integral 16	12.8533	1.5270
Integral 17	28.9029	1.2531
Integral 18	3.9260	0.6828

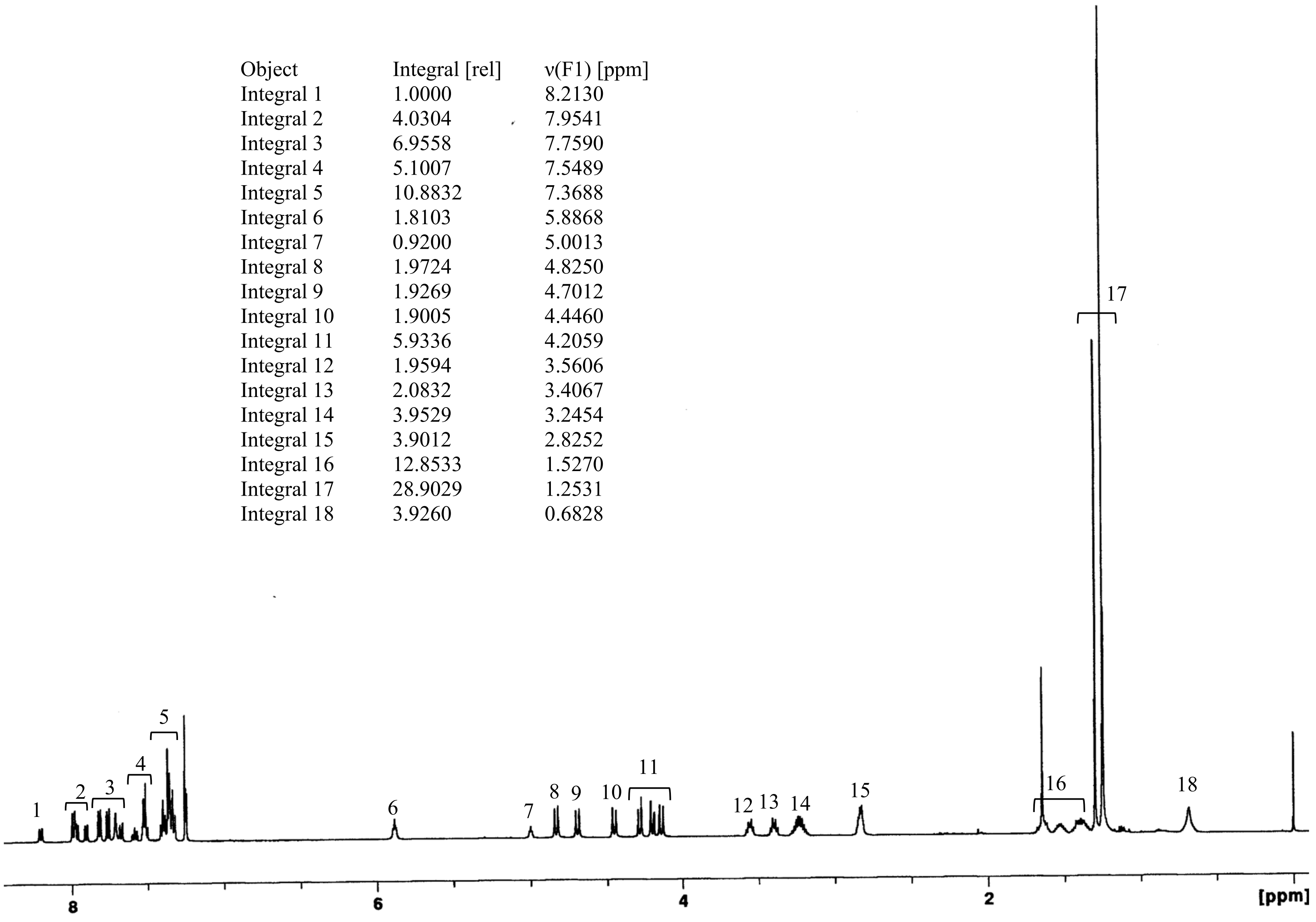

Figure S13. ^{1}H NMR spectrum (500 MHz, $CDCl_3$, rt) of Napht urea **4a**.

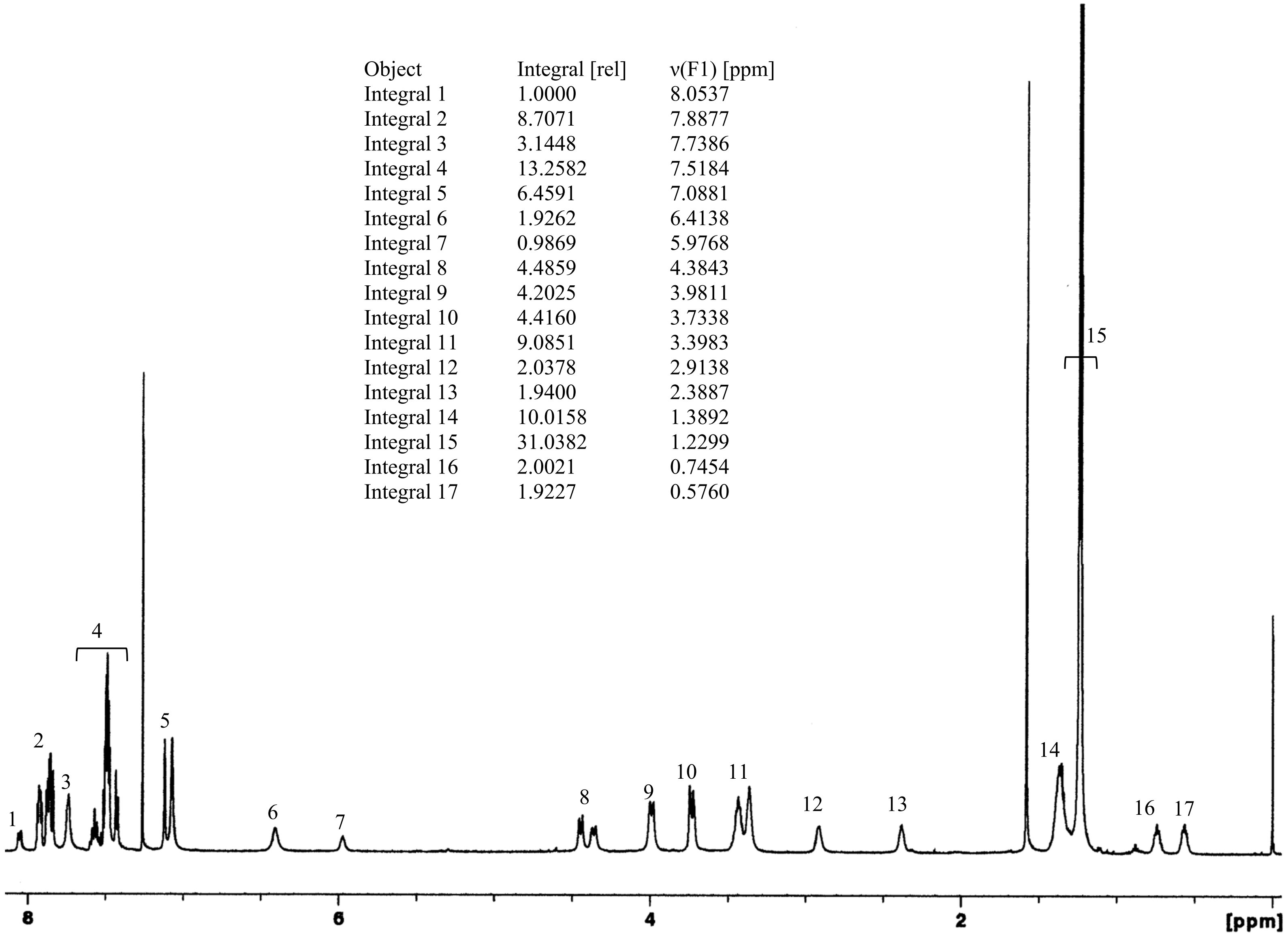

Figure S14. ^{1}H NMR spectrum (500 MHz, $CDCl_3$, rt) of Napht thiourea **4b**.

Object	Integral [rel]	ν(F1) [ppm]
Integral 1	1.0000	8.5643
Integral 2	1.0684	8.4207
Integral 3	5.7522	8.1840
Integral 4	3.6971	8.0327
Integral 5	5.7029	7.9008
Integral 6	5.0337	7.7727
Integral 7	5.9525	7.6446
Integral 8	5.6341	7.4506
Integral 9	1.8902	5.9605
Integral 10	1.1325	5.0253
Integral 11	3.7578	4.8545
Integral 12	2.0625	4.5014
Integral 13	2.0459	4.3113
Integral 14	3.8461	4.1754
Integral 15	2.0270	3.6361
Integral 16	4.0547	3.4498
Integral 17	2.2545	3.2092
Integral 18	3.8572	2.8871
Integral 19	2.1591	1.7889
Integral 20	4.1977	1.4862
Integral 21	26.5744	1.3039
Integral 22	3.5442	0.7373

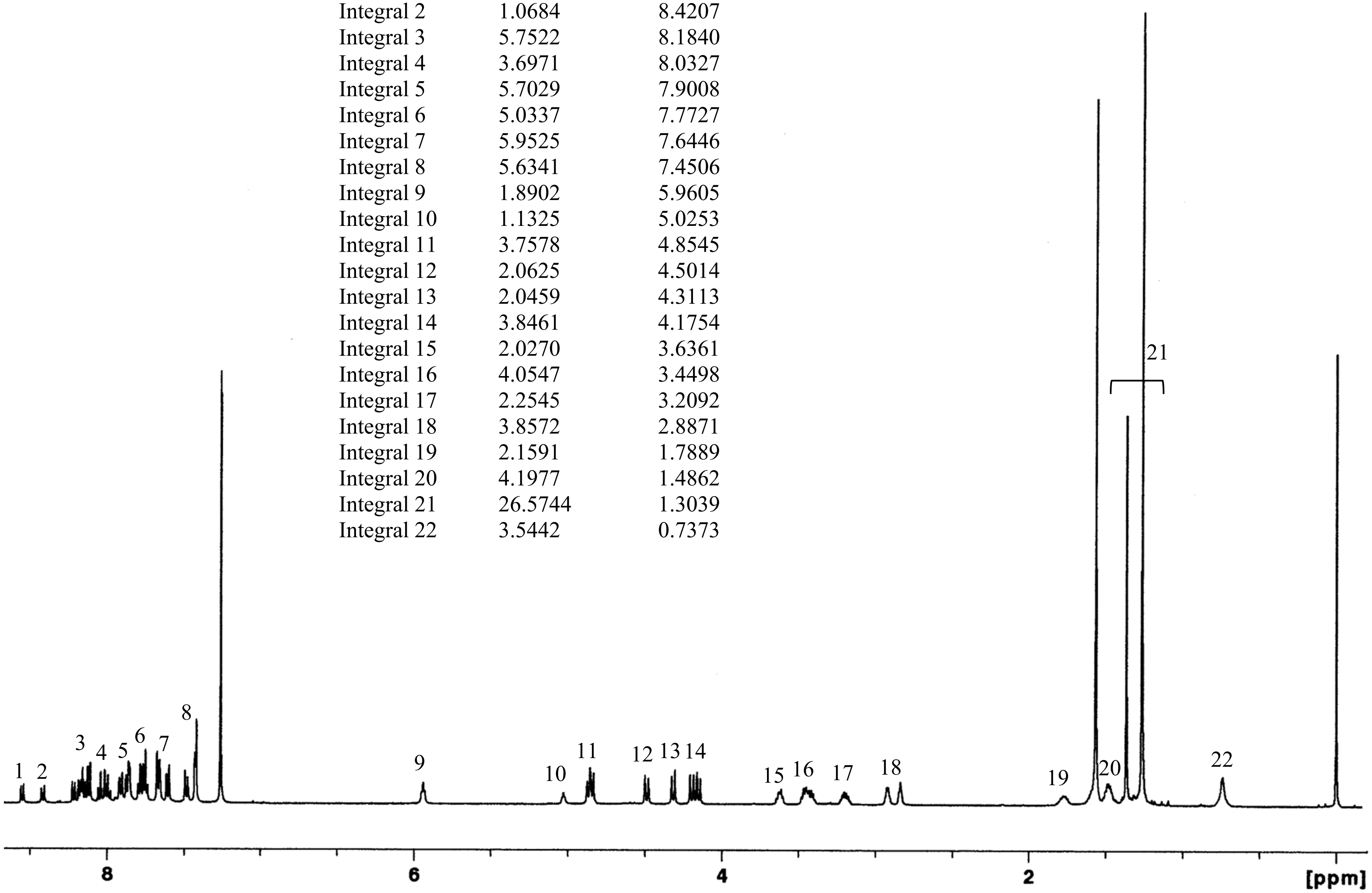

Figure S15. ^{1}H NMR spectrum (500 MHz, $CDCl_3$, rt) of Pyr urea **4c**.

Peak	ν(F1) [ppm]	Peak	ν(F1) [ppm]
1	157.2039	23	125.7901
2	156.0722	24	124.4283
3	155.2836	25	121.2672
4	146.5784	26	120.8503
5	146.3082	27	119.7786
6	134.4158	28	75.2743
7	134.2276	29	74.7646
8	133.9107	30	68.4530
9	130.2922	31	66.2720
10	130.1052	32	63.9691
11	129.3864	33	40.0234
12	128.8173	34	34.3321
13	128.7306	35	34.3085
14	128.4983	36	31.5204
15	127.6440	37	31.4172
16	127.5943	38	27.7708
17	127.5450	39	27.8040
18	127.4307	40	26.2627
19	126.1825	41	25.0135
20	126.1487		
21	125.9774		
22	125.8742		

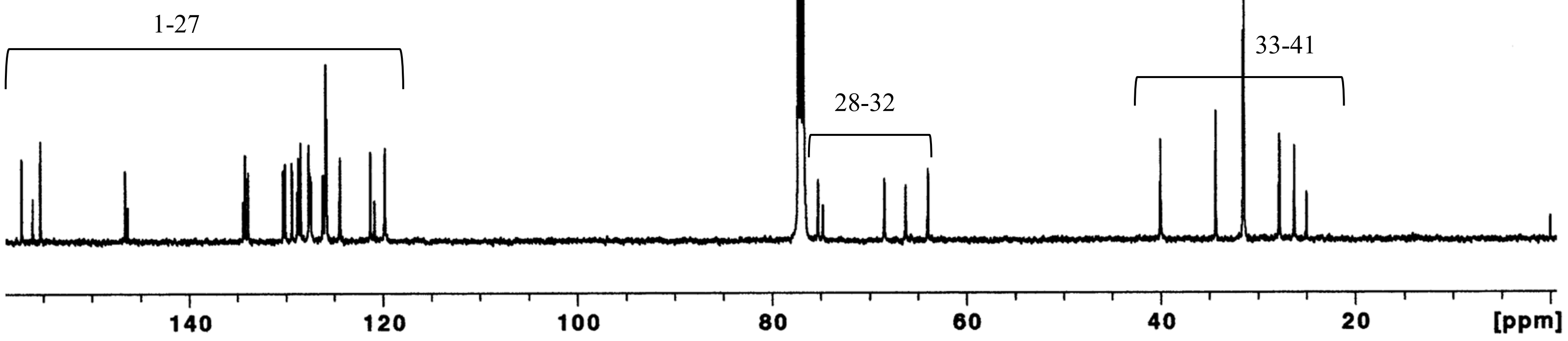

Figure S16. ^{13}C NMR spectrum (125.8 MHz, $CDCl_3$, rt) of Napht urea **4a**.

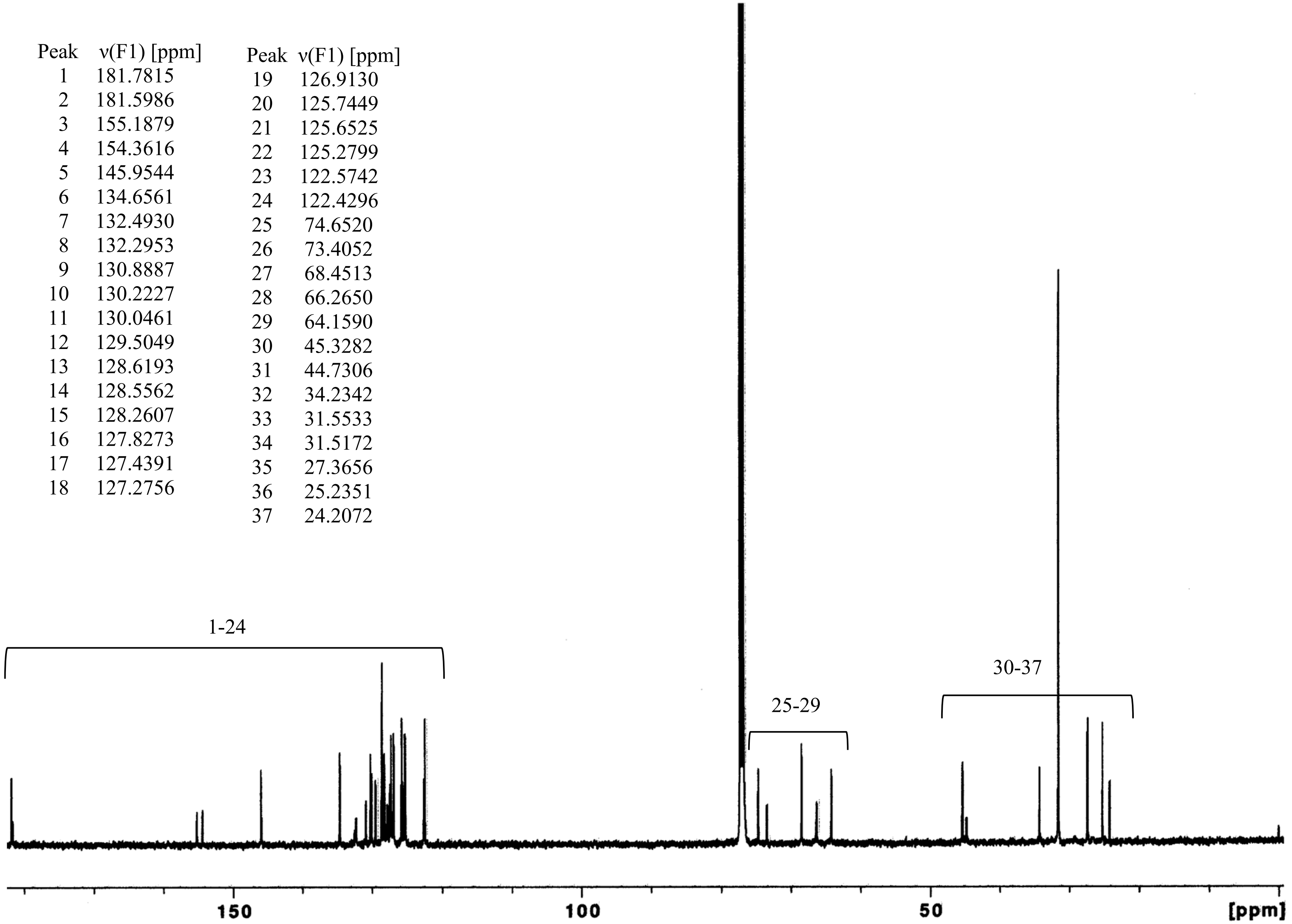

Peak	ν(F1) [ppm]	Peak	ν(F1) [ppm]
1	181.7815	19	126.9130
2	181.5986	20	125.7449
3	155.1879	21	125.6525
4	154.3616	22	125.2799
5	145.9544	23	122.5742
6	134.6561	24	122.4296
7	132.4930	25	74.6520
8	132.2953	26	73.4052
9	130.8887	27	68.4513
10	130.2227	28	66.2650
11	130.0461	29	64.1590
12	129.5049	30	45.3282
13	128.6193	31	44.7306
14	128.5562	32	34.2342
15	128.2607	33	31.5533
16	127.8273	34	31.5172
17	127.4391	35	27.3656
18	127.2756	36	25.2351
		37	24.2072

Figure S17. ^{13}C NMR spectrum (125.8 MHz, $CDCl_3$, rt) of Napht thiourea **4b**.

Peak	ν(F1) [ppm]	Peak	ν(F1) [ppm]
1	157.4760	21	124.6595
2	155.2484	22	124.5627
3	146.6176	23	124.2002
4	130.9646	24	124.1255
5	130.4381	25	121.4981
6	130.3122	26	120.5163
7	130.0907	27	120.3046
8	129.4014	28	75.2079
9	128.8027	29	74.6833
10	127.6971	30	68.5188
11	127.4939	31	66.3923
12	127.3737	32	64.0306
13	126.9173	33	40.0514
14	126.7946	34	34.3335
15	126.6727	35	31.5642
16	126.1898	36	31.4456
17	125.6253	37	27.8234
18	125.4019	38	27.7078
19	125.2736	39	26.3652
20	124.8015	40	25.0059

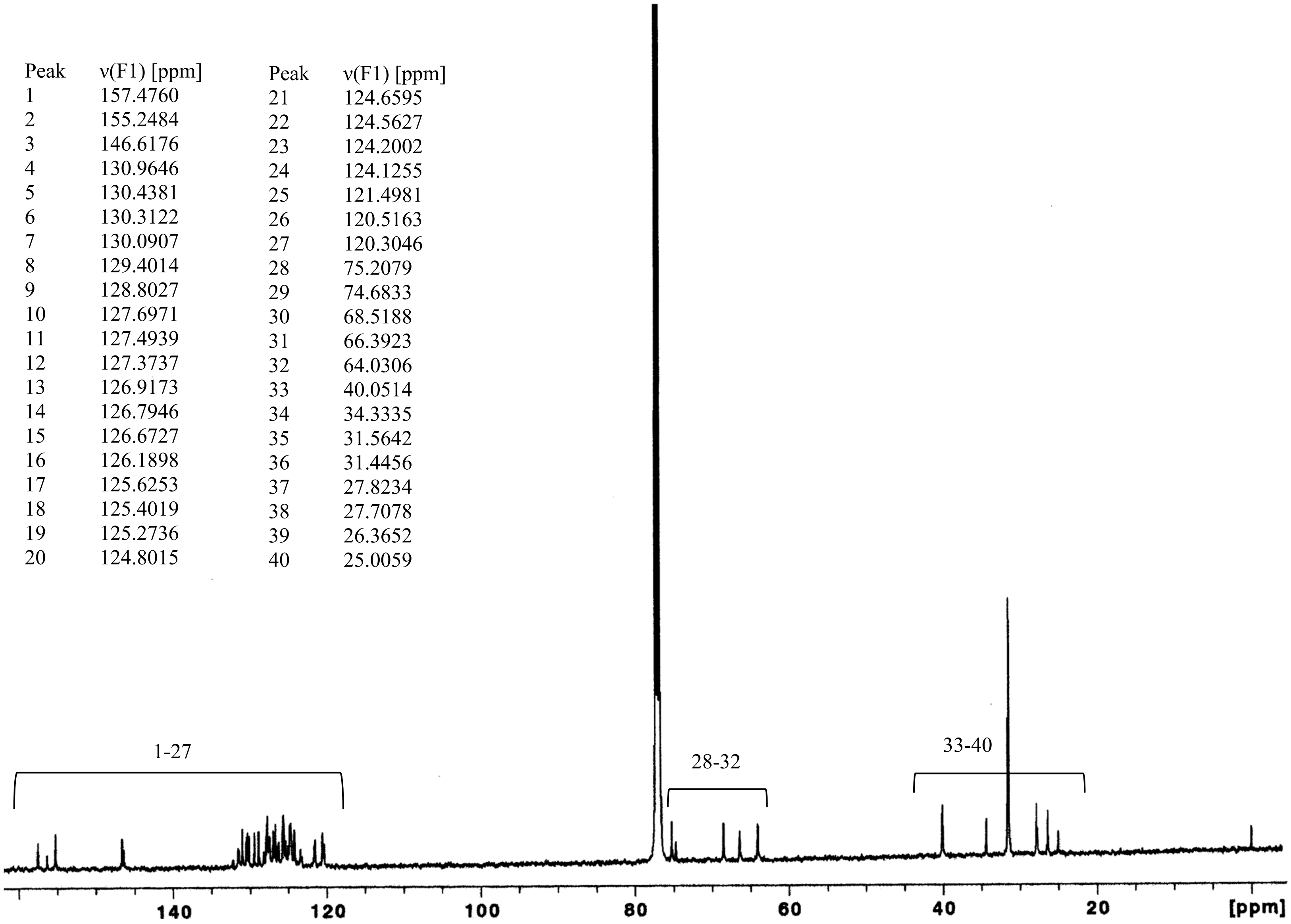

Figure S18. ^{13}C NMR spectrum (125.8 MHz, $CDCl_3$, rt) of Pyr urea **4c**.

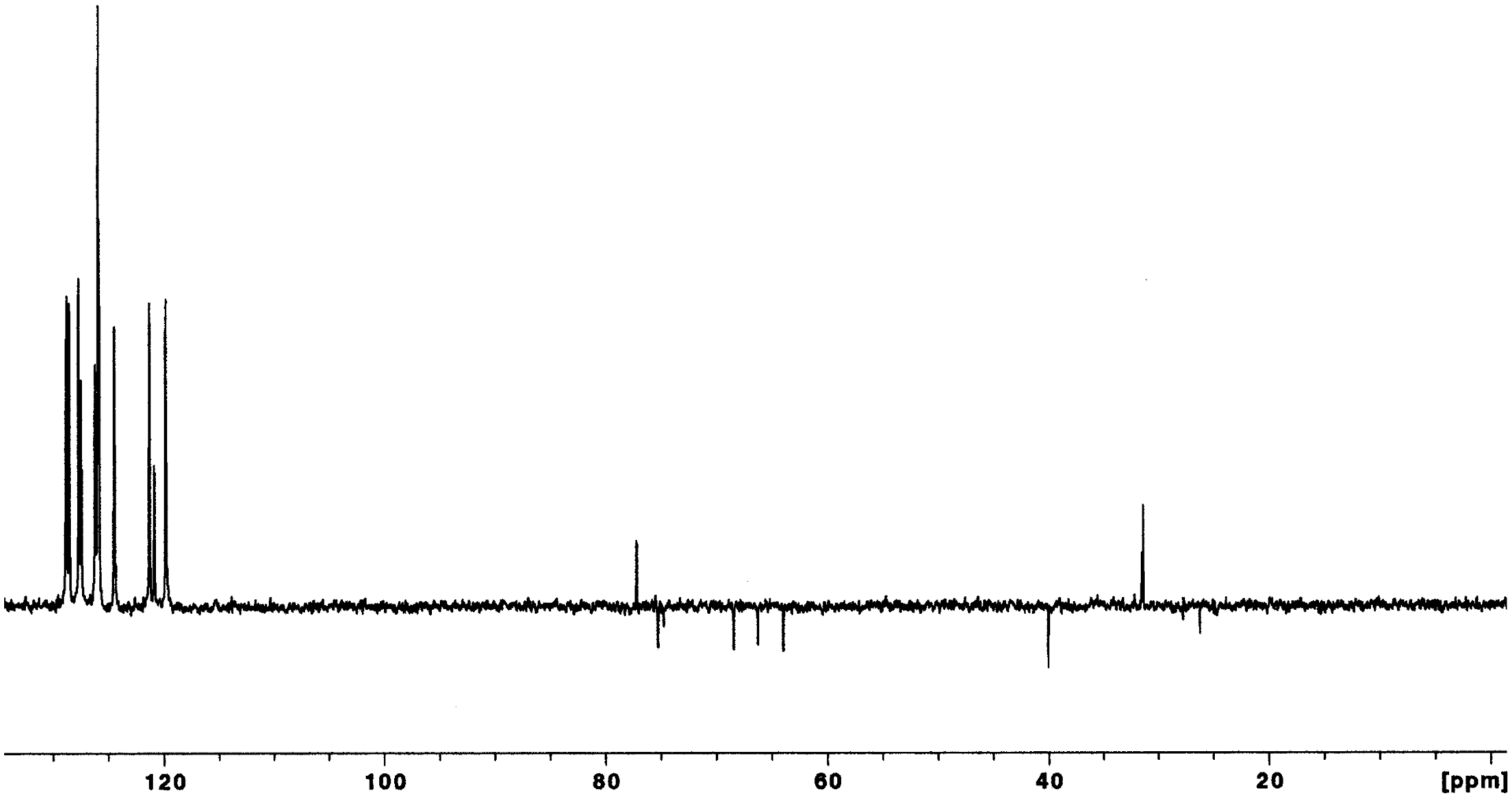

Figure S19. DEPT spectrum (125.8 MHz, $CDCl_3$, rt) of Napht urea **4a**.

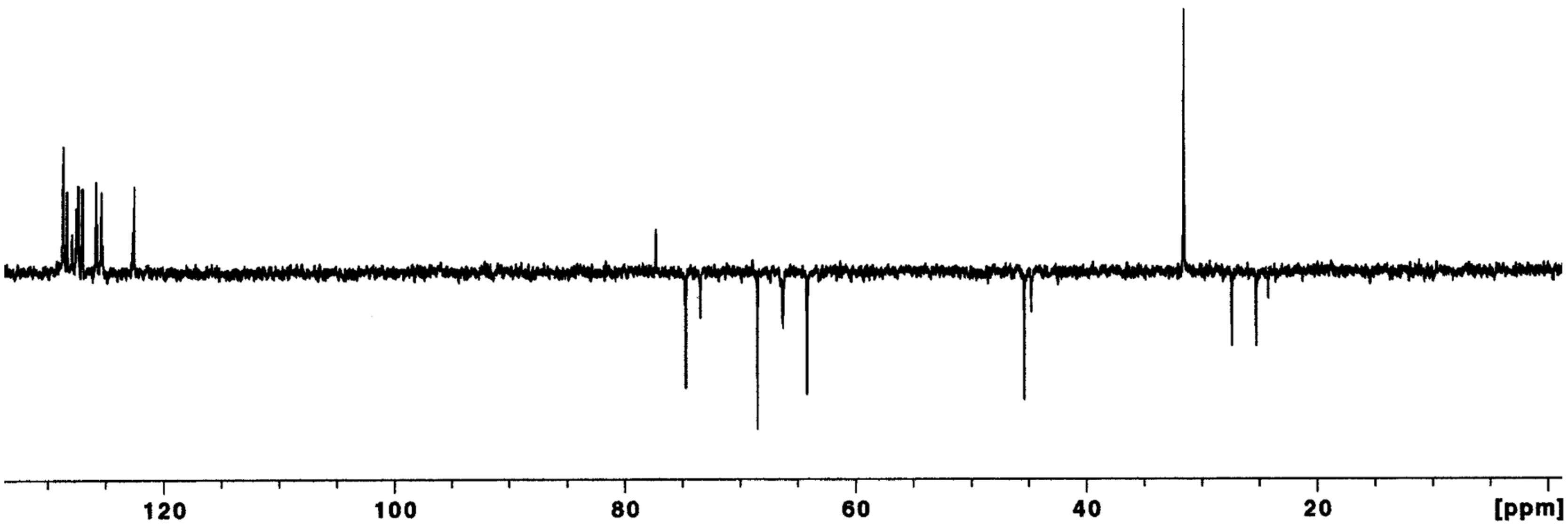

Figure S20. DEPT spectrum (125.8 MHz, $CDCl_3$, rt) of Napht thiourea **4b**.

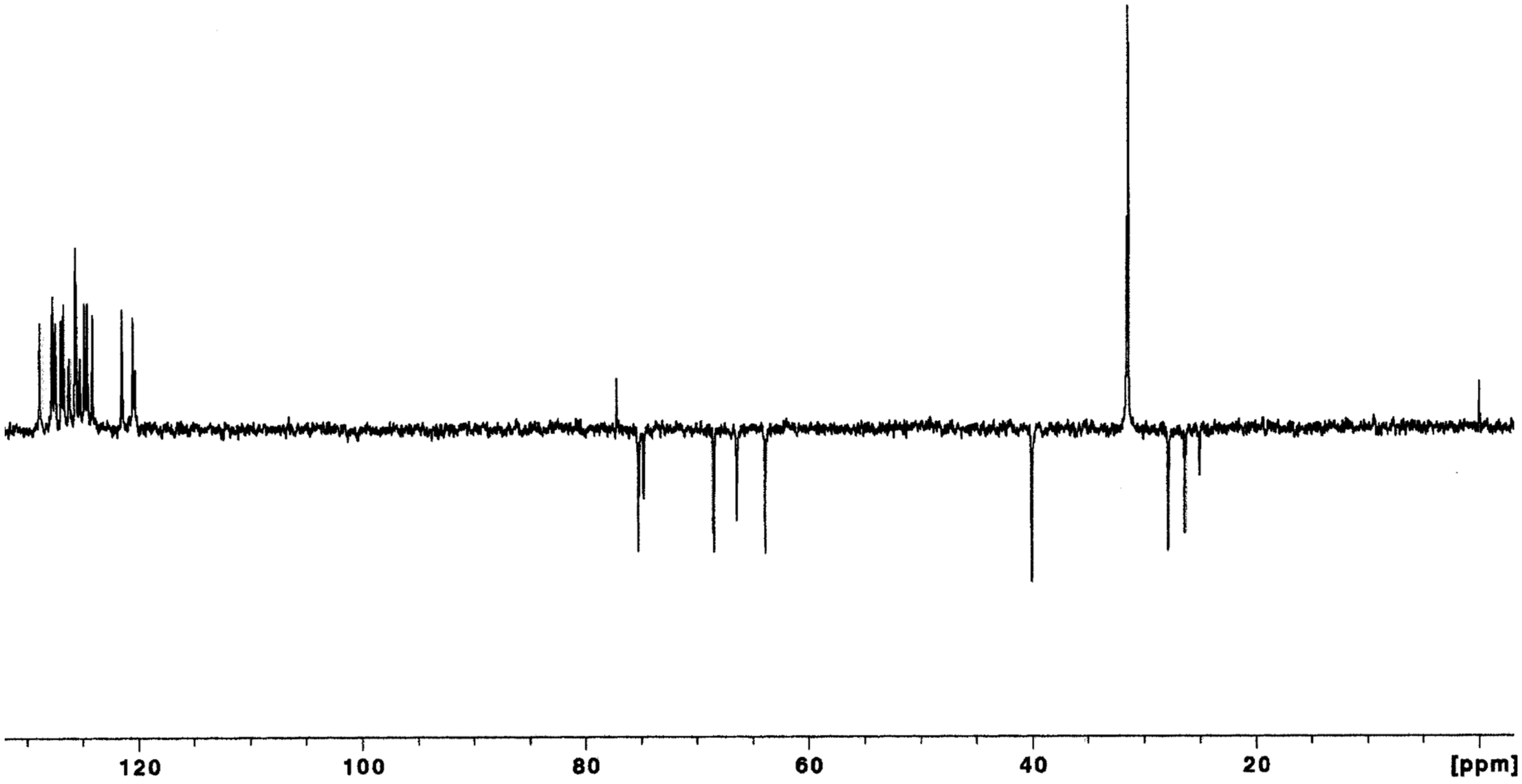

Figure S21. DEPT spectrum (125.8 MHz, $CDCl_3$, rt) of Pyr urea **4c**.

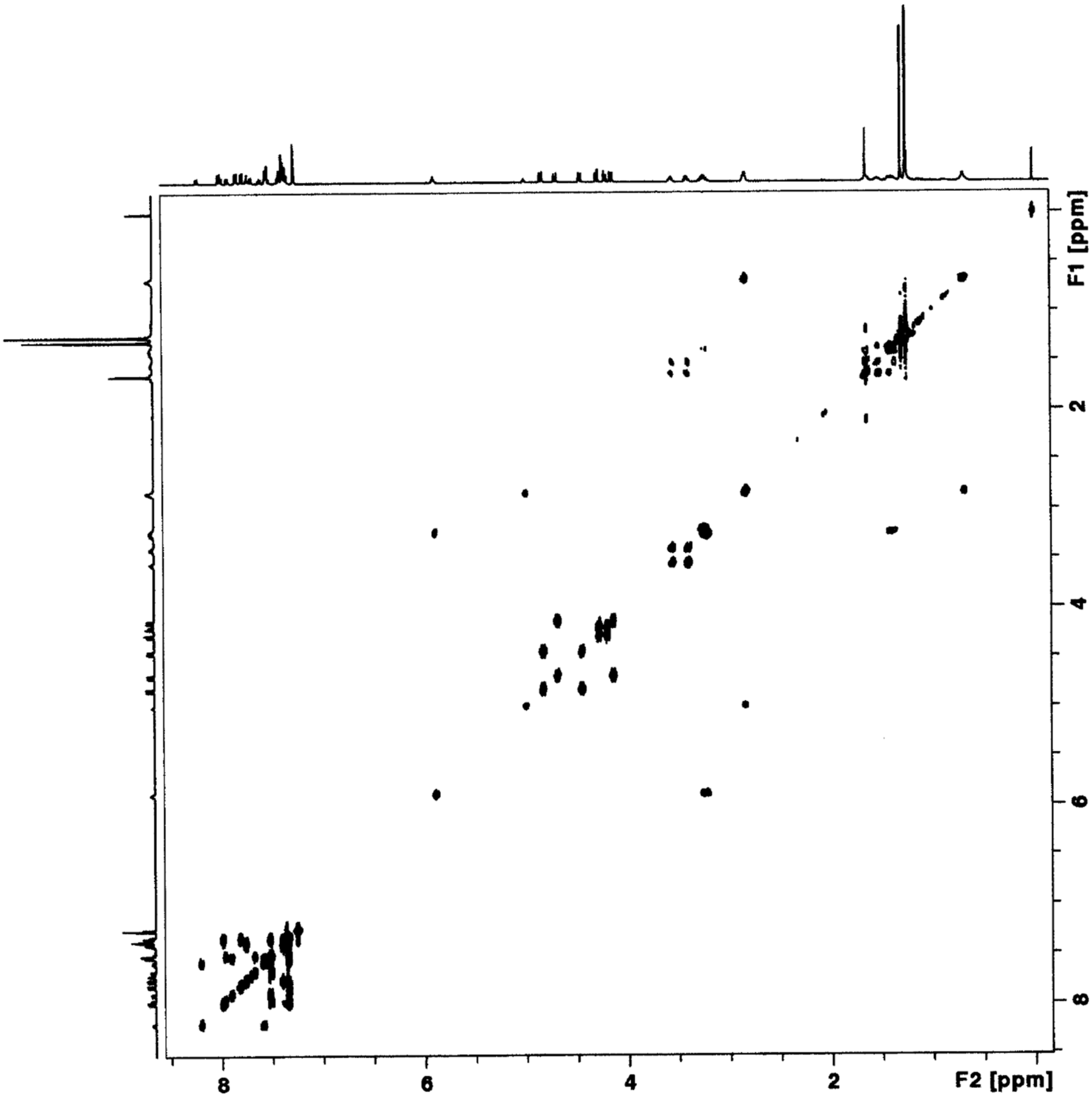

Figure S22. COSY spectrum (500 MHz, $CDCl_3$, rt) of Napht urea **4a**.

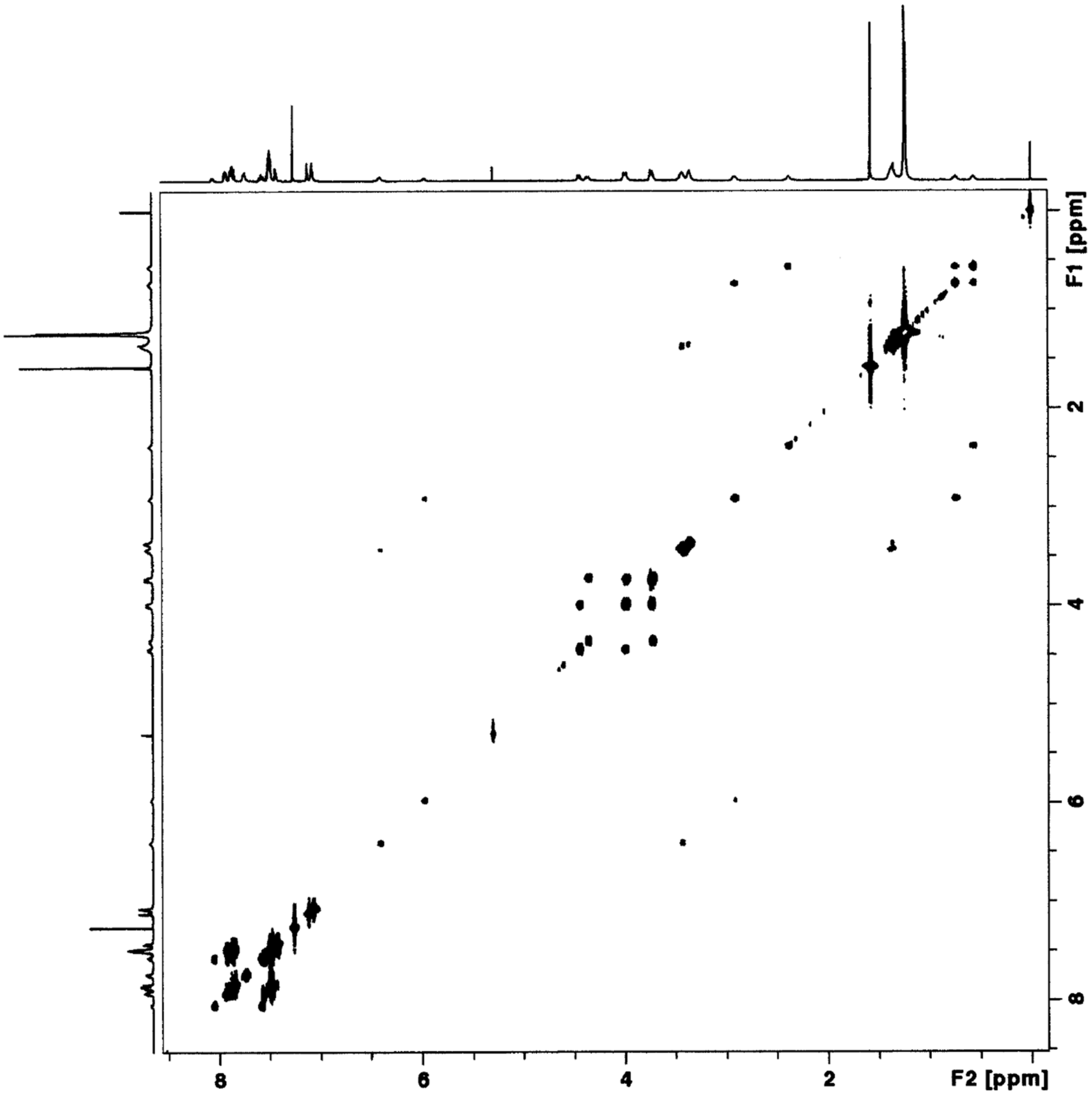

Figure S23. COSY spectrum (500 MHz, $CDCl_3$, rt) of Napht thiourea **4b**.

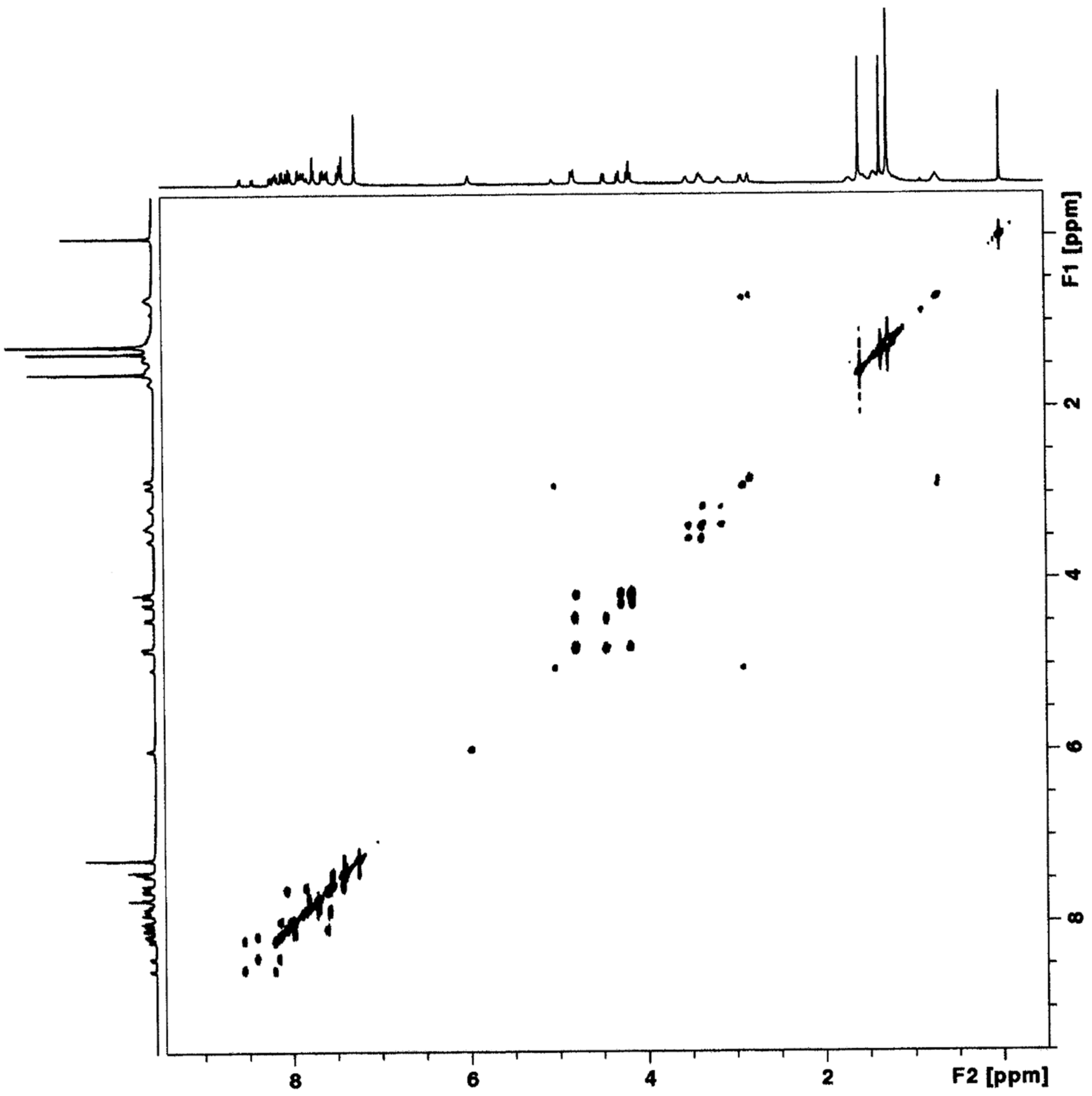

Figure S24. COSY spectrum (500 MHz, $CDCl_3$, rt) of Pyr urea **4c**.
